# Roquin2 suppresses breast cancer progression by inhibiting tumor angiogenesis via selectively destabilizing proangiogenic factors mRNA

**DOI:** 10.7150/ijbs.59891

**Published:** 2021-07-13

**Authors:** Meicen Zhou, Wenbao Lu, Bingwei Li, Xiaochen Yuan, Mingming Liu, Jianqun Han, Xueting Liu, Ailing Li

**Affiliations:** 1 Department of Endocrinology, Beijing Jishuitan Hospatial, The 4 th Clinical Medical College of Peking University, Beijing, 100035, China; 2 Institute of Microcirculation, Chinese Academy of Medical Sciences & Peking Union Medical College, Beijing, 100005, China

**Keywords:** Roquin2, tumor angiogenesis, metastasis, mRNA degradation, breast cancer

## Abstract

Tumor angiogenesis is an essential step in tumor growth and metastasis. The initiation of tumor angiogenesis is dictated by a shift in the balance between proangiogenic and antiangiogenic gene expression programs. Roquin2 is a zinc-finger RNA-binding protein with important roles in mediating the expression of inflammatory genes, such as *TNF*, *IL6* and *PTGS2*, which are also important angiogenic factors. In this study, we demonstrate that Roquin2 functions as a potent tumor angiogenesis regulator that inhibits breast tumor-induced angiogenesis by selectively destabilizing mRNA of proangiogenic gene transcripts, including *endoglin* (*ENG*)*, endothelin-1* (*EDN1*)*, vascular endothelial growth factor B* (*VEGFB*) and* platelet derived growth factor C* (*PDGFC*). Roquin2 recognizes and binds the stem-loop structure in the 3'untranslated region (3'UTR) of these mRNAs via its ROQ domain to destabilize mRNA. Moreover, we found that Roquin2 expression was reduced in breast cancer cells and tissues, and associated with poor prognosis in breast cancer patients. Overexpression of Roquin2 inhibited breast tumor-induced angiogenesis *in vitro* and *in vivo*, whereas silencing Roquin2 enhanced tumor angiogenesis. *In vivo* induction of Roquin2 by adenovirus significantly suppressed breast tumor growth, metastasis and angiogenesis. Taken together, our results identify that Roquin2 is a novel breast cancer suppressor that inhibits tumor angiogenesis by selectively downregulating the expression of proangiogenic genes.

## Introduction

Tumor angiogenesis is a key step for further growth and distal metastasis of solid tumors 1,2. However, the underlying molecular mechanism of tumor angiogenesis is not fully understood. Tumor cells activate vascular endothelial cells by secreting certain angiogenic factors thereby contributing to the formation of microvascular networks inside the tumor tissue 3. These angiogenic factors aid in the communication between tumor cells and vascular cells 4. Angiogenic factors could be classified into proangiogenic factors (e.g., PDGFs 5, VEGFs 6, EDN1 7, ENG 8) and antiangiogenic factors (e.g., tissue inhibitor of metalloproteinases 9, angiopoietin like 4 10, serpin family F member 1 11). The balance between these factors determines whether angiogenesis is induced or inhibited 12. Angiogenesis is mostly initiated by either an increase in the expression of proangiogenic factors or a decrease in the expression of anti-angiogenesis factors 13. Although recent research has broadened our understanding of tumor angiogenesis mechanisms, how angiogenesis-related genes dysregulation develops in cancer cells through posttranscriptional mechanisms, especially by RNA-binding proteins, remains unclear.

Roquin2 is an RNA-binding protein belonging to the Roquin family (consisting of Roquin1 and Roquin2) and encoded by the *Rc3h2* gene 14. Both Roquin1/2 have a RING domain, a conserved ROQ domain, and a zinc finger domain in the N-terminal 15. It is believed that the ROQ domain binds to the conserved stem-loop motif in the 3'UTR of mRNAs to promote degradation of the target mRNAs 16-20. Roquin2 can recruit CCR4-CAF-NOT complex and induce mRNA deadenylation and degradation 21. Both Roquin2 and Roquin1 can also regulate the differentiation of follicular helper T cells by degrading the mRNAs of co-stimulators inducible T cell costimulatory (Icos) and Ox40 22. In addition, Roquin2 can modulate the differentiation of NKT cells 23 and stress reactions via ubiquitination-mediated degradation of apoptosis signal-regulating kinase 1 (ASK1) 24. Therefore, Roquin2 is a key negative regulator in inflammation. Most Roquin2-deficient mice would die within several days after birth 25. It was recently reported that Roquin2 could be targeted and degraded via protein dephosphorylation by tumor suppressors, Kelch-like protein 6 (KLHL6) 26 and protein tyrosine phosphatase non-receptor type 14 (PTPN14) 27 in B cell cancers. However, the role and the underlying mechanism of Roquin2 in cancer progression and especially in solid tumor angiogenesis remain unclear.

In this study, we identified Roquin2 as a tumor suppressor. Roquin2 inhibits tumor angiogenesis by selectively destabilizing proangiogenic gene transcripts, including *ENG*, *EDN1*, *VEGFB*, and *PDGFC*. Roquin2 expression was decreased in breast tumor tissues and cells. Ectopic expression of Roquin2 in tumor cells inhibited tumor angiogenesis, tumor growth and metastasis *in vitro* and *in vivo*, whereas further silencing Roquin2 expression using shRNAs promoted tumor angiogenesis. Analyzing public breast cancer databases 28 revealed that low levels of Roquin2 strongly correlated with poor survival of breast cancer patients. Notably, Roquin2 expression negatively correlated with the expression of proangiogenic factors, including *ENG*, *EDN1*, *VEGFB*, *platelet endothelial cell adhesion molecule-1* (*PECAM1*), *Angiogenin* (*ANG*), *tyrosine kinase with immunoglobulin like and EGF like domains 1* (*Tie1*), and *EphB4 type-B receptor 4* (*EPHB4*) in human breast tumors. These findings demonstrate that Roquin2 acts as a potent breast tumor suppressor and is involved in regulating tumor angiogenesis pathway by suppressing the gene expression of proangiogenic factors.

## Materials and methods

### Cell lines and plasmids

Human breast cancer cell lines (MDA-MB-231, MDA-MB-468, and MCF7), human normal mammary epithelial cell lines (MCF-10A, MCF-12A), and human lung cancer cell line A549 were obtained from the American Type Culture Collection (ATCC) and cultured in DMEM or PRM1640 medium with 10% FBS plus 1% Peni/Stro, respectively. Human hepatocellular carcinoma cells SMMC-7721 was purchased from the Cell Bank of Shanghai Institute of Cell Biology, Chinese Academy of Sciences (Shanghai, China), and cultured in PRM1640 with 10% FBS plus 1% Peni/Stro. Immortalized human umbilical vein endothelial cells (HUVECs), HEK293, and HEK293T cells were obtained from National Infrastructure of Cell Line Resource (Beijing, China) and cultured in Endothelial Cell Medium (Cat #1001, ScienCell) or DMEM medium with 10% FBS plus 1% Peni/Stro, respectively. The human full-length Roquin2 coding sequence (NM_001100588.1) was synthesized, sequenced and inserted into pEGFP-N1 vector at EcoR I and Age I sites. Roquin2 serial deletion plasmids were generated by inserting the PCR-amplified fragments into pEGFP-N1 vector at EcoR I and Age I sites. A set of luciferase reporters were constructed by inserting the full-length 3'UTRs of human *PDGFC*, *VEGFB*, *EDN1*, and *ENG* into the pGL3 control vector (Promega) between Xba I and Fse I sites, respectively. For stem-loop deletion reporters, point mutated and truncated *PDGFC*-3'UTR (∆stem-loop) and *EDN1*-3'UTR (∆stem-loop) were amplified, sequenced, and inserted into pGL3 control vector using Phusion Site-Directed Mutagenesis Kit (Thermo Scientific). For stem-loop insertion constructs, the stem-loop sequences of *PDGFC* and* EDN1* 3'UTRs were inserted into pGL3-β-actin^3'UTR^ reporter at 555 base pair, respectively.

### Antibodies and Reagents

Polyclonal rabbit anti-Roquin2 (ab99090), anti-GFP (ab290), and normal rabbit IgG (ab37415) were from Abcam. Rabbit anti-PDGFC (55076-1-AP), ENG (10862-1-AP), EDN1 (12191-1-AP) antibodies were from Proteintech. Rabbit anti-VEGFB (#2463) antibody was purchased from Cell Signaling Technology. Anti-CD31 (sc-376764) antibody was obtained from SantaCruz Biotechnology. Monoclonal β-actin (A2066) antibody was from Sigma Company. Thiazolyl Blue Tetrazolium Bromide (MTT) (M2128), actinomycin D (ActD, A1410), and 5, 6-Dichlorobenzimidazole 1-β-D-ribofuranoside (DRB) (D1916) were from Sigma-Aldrich (St. Louis, USA). G418 (G8168) and puromycin (P8833) were also from Sigma. Protein A/G PLUS-Agarose beads were from Sant Cruz Biotechnology. GFP-coated beads (GFP-Trap Dynabeads, GTD20) were from ChromoTek (Germany). GFP/Roquin2-expressing adenoviruses and GFP control adenoviruses were packaged and purified at GeneChem (Shanghai, China).

### RNA isolation, qRT-PCR and PCRArray

Total RNA was extracted with TRIzol from tissues or cultured cells and reverse transcribed to cDNA for qPCR using SYBR green Fast Master Mix (Roche). Gene expression level was based on the ∆∆Ct method and normalized to GAPDH. The QIAGEN Human Angiogenesis PCRArray Kit was used to analyze angiogenesis-related gene expression. Total RNA was extracted with TRIzol from MDA-MB-468/Roquin2 and MDA-MB-468/GFP cells, purified using the RNA PureLink Kit (Thermo Fisher Scientific), reverse transcribed to cDNA, and added to each well of the PCRArray plates combined with SYBRGreen qPCR MasterMix according to the manufacturer's instructions. Data analysis was based on the ΔΔCt method, with normalization to three different housekeeping genes. The primer sequences for qPCR and PCR were listed in Supplementary Table S3.

### Western Blotting

Cell or tissue samples were collected and lysed with a modified RIPA buffer containing PMSF and protease inhibitor cocktail (Roche, Switzerland). Protein concentration was measured by BCA method. Equal amount of protein lysates was subjected to electrophoresis by SDS-PAGE and transferred onto a polyvinylidene membrane. The membrane was then blocked with 5% fat-free milk and incubated with primary antibody overnight at 4℃. The band was detected with HRP-conjugated secondary antibodies using ECL chemiluminescent detection method.

### Wound healing assay

1 x 10^4^ HUVECs were seeded in each well of 96-well plate in triplicates and incubated at 37 ℃. After the cells forming a monolayer, a scratch of the cell monolayer was created using a pipette tip and the cells were incubated for 24 hours. The images of the scratch were acquired using a microscope (Zeiss, Germany) and the distance of the wound was calculated and the open area was measured.

### Tube formation assay

The tumor cell culture medium was changed to serum-free RPMI-1640 or DMEM medium for 48 h and then collected, centrifuged and filtered to obtain tumor conditioned medium (CM). The wells of the 96-well plate were coated with 50µl pre-thawed Matrigel (BD Biosciences, USA) and incubated for 1 h in a 37 ℃ incubator. 1 x 10^4^ HUVECs were seeded on the gel with 200µl of CM concentrated using an ultrafiltration device (Millipore, USA). The tube formation of HUVECs was observed after incubated 12 h using a microscope (Zeiss, Germany).

### RNA-sequencing analysis

MDA-MB-468/Roquin2, MCF7/Roquin2, A549/Roquin2, SMMC-7721/Roquin2 cell and their control cell (expressing GFP) were cultured for 36 h, and total RNA was extracted using Trizol method. The integrity of the cleaned RNA was confirmed by NanoDrop spectrophotometry. RNA sequencing was completed by the Allwegene Technology Inc. in Beijing. The cDNA library was then constructed using PCR amplification. RNA-seq was performed with the PE150 sequencing strategy by the Illumina second-generation high-throughput sequencing platform. RNA-seq reads with inferior quality or Adapters were filtered. Clean reads data were processed using Tophat2 and Cufflinks software to complete the alignment of transcriptomes. Genes not expressed in any sample were excluded from further analysis. Differentially expressed genes and transcripts were then filtered for FDR adjusted p-values less than or equal to 0.05. RNA-seq data were deposited in NCBI SRA (https://www.ncbi.nlm.nih.gov/sra) (PRJNA668641).

### RNA immunoprecipitation (RIP)

RIP experiment was conducted as previously described 29. The protein-RNA complexes were immunoprecipitated by protein A/G beads, and total RNA extracted with TRIzol, followed by detection with RT-PCR.

### mRNA stability

Roquin2/GFP was expressed in MDA-MB-231 cells, and then actinomycin D (ActD, 5µg/mL) and 5,6-Dichlorobenzimidazole riboside (DRB, 5µg/mL) were added to block *de novo* RNA synthesis. Total RNA was collected at different time points and relatively mRNA level was analyzed by qPCR. Half-life of the mRNA was determined by comparing with the levels of mRNA before adding ActD and DRB. The half-lives of different genes in Roquin2 knockdown cells and their corresponding pScrambled control cells were also tested as described above.

### Luciferase reporter assays

Luciferase assay was performed as described previously 29. pGL3 luciferase reporter constructs containing full-length or segment of 3'UTR of different genes were transfected into HEK293 cells along with GFP-Roquin2, aa 1-410, aa 410-1191, aa 171-325, and GFP-control constructs, respectively. All transfections were conducted in triplicate and repeated at least three times. The luciferase activity was measured 36 hrs after transfection using a Dual-Luciferase Reporter Assay System (Promega, USA).

### shRNA lentivirus

Two lentiviral shRNAs (NM_018835.2-475s21c1; NM_018835.2-725s21c1) targeting human Roquin2 mRNA and two lentiviral shRNAs targeting human PDGFC (NM_016205.1-831s1c1) and EDN1 (NM_001955.x-677s1c1) were purchased from Sigma. A scramble control shRNA was used as a control. Lentiviral particles were packaged in HEK293T cells by cotransfecting shRNA-pLKO.1, pCMV-dR8.2, and pMD2.G constructs. After two rounds infection, the target cells were selected with puromycin (2.5µg/mL) for two weeks, followed by further study.

### Animal Study

6-8 weeks female BALB/c nude mice were bought from the Institute of Laboratory Animal Science, Chinese Academy of Medical Sciences (CAMS) & Peking Union Medical College (PUMC). The mice were bred in cages with filter tops in a laminar flow hood in pathogen-free condition, with a 12 h light, 12 h dark cycle. All experimental procedures were approved by the Experimental Animal Care and Ethics Committee of the Institute of Microcirculation, CAMS & PUMC. MDA-MB-468/Roquin2-GFP (5 × 10^6^/100µL PBS), MDA-MB-231/Roquin2-GFP (3 × 10^6^/100µL PBS) and their control cells were injected subcutaneously into the abdominal mammary glands of nude mice, respectively. Tumor sizes were measured and recorded for drawing tumor growth curve. For tumor treatment with adenovirus, MDA-MB-231cells (3 × 10^6^/100µL PBS) were injected into nude mice aged 6-8 weeks according to above methods. When tumors reached approximately 5-7 mm in diameter, GFP/Roquin2-expressing adenoviruses or GFP-expressing control adenoviruses (packaged at GeneChem, Shanghai) were injected into the tumors five times with a 10^10^ pfu/tumor each time. Tumor size was measured by the formula length × width × high (mm^3^).

### Tumor tissue samples

21 human breast cancer samples and their matched surrounding 'healthy' tissues were obtained between October 2018 and January 2021 from Beijing Jishuitan Hospatial, the 4^th^ Clinical Medical College of Peking University. The tumors have mixture molecular subtypes, including eight luminal A, three luminal B, and seven triple negative breast cancers. The subtypes of the rest three tumors are unknown. All subjects gave their informed consent for inclusion before they participated in the study. The study was conducted in accordance with the Declaration of Helsinki, and the experimental protocols were approved by the Ethics Committee of Beijing Jishuitan Hospatial, the 4^th^ Clinical Medical College of Peking University and the Medical Ethics Committee of the Institute of Microcirculation, CAMS & PUMC.

### ELISA assay

Human PDGFC and EDN1 levels of the CMs from tumor cells was assessed with commercially available ELISA kits (EH365RB, Invitrogen; 355377, USBiological, USA) according to the manufacturer's instructions, respectively.

### RNA-ChIP

RNA-ChIP assay was performed as previously 29. Briefly, Roquin2/GFP fusion protein was expressed in MDA-MB-468 cells for 30 h. Then, cells were cross-linked for 10 min by addition of formaldehyde (1% v/v). Glycine was used to stop crosslinking (125 mM). Cells were washed with cold PBS, resuspended in 500 μL of polysome lysis buffer, and placed on ice for 5 min. Cell lysates were collected by centrifugation at 4℃, and re-suspended in 500 μL of polysome lysis buffer. The lysates were sonicated and pre-clear with rabbit IgG to remove non-specific background. Pre-cleared lysates were used for IP with GFP-coated beads or rabbit IgG-coated beads at 4℃. After pull-down, 100 μL supernatants were taken out for Input. Each immune complex was washed five times with ice-cold NT2 buffer. RNA was isolated with Trizol, and re-suspended in 50 μL of RNase-free water, followed by DNase I treatment and further detection.

### Sequence Alignments and Stem-loop Structure Prediction

For 3'UTR stem-loop structure sequence conservation analysis of *PDGFC*, *VEGFB*, *EDN1*, and *ENG*, the 3'UTR sequences were extracted for different species from the National Center for Biotechnology Information (NCBI) database: *PDGFC* 3'UTR: human (*Homo sapiens*; accession number NM_016205.3), chimpanzee (*Pan troglodytes*; XM_001140766.6), mouse (*Mus musculus*; XM_017319655.2); sand rat (*Heterocephalus*; XM_004868546.3), Spalacidae (*Nannospalax*, XM_017803542.2); *VEGFB* 3'UTR: human (*Homo sapiens*; NM_003377.5), chimpanzee (*Pan troglodytes*; XM_001144738.6), mouse (*Mus musculus*; NM_011697.3), Saimiri (*Saimiri boliviensis*, XM_039470476.1); *EDN1* 3'UTR: human (*Homo sapiens*; NM_001168319.2), chimpanzee (*Pan troglodytes*; XM_009450521.3), mouse (*Mus musculus*; NM_010104.4), rat (*Rattus*; NM_012548.2); *ENG* 3'UTR: human (*Homo sapiens*; NM_001114753.2), mouse (*Mus musculus*; NM_007932.2), chimpanzee (*Pan paniscus*; XM_016961724.1), rat (*Rattus*; NM_001010968.2). Stem-loop sequence conservation analysis was performed using DNAMAN software. The stem-loop structure was predicted through RNAfold web server.

### Statistical analysis

Data in bar graphs represent mean ± SEM of at least three biological repeats. Statistical analysis was performed using Student's *t*-test by comparing treatment versus vehicle control or otherwise as indicated. P-value < 0.05 was considered to be statistically significant.

## Results

### Roquin2 is downregulated in breast cancer tissues and cells and associated with poor survival

**Figure 1:**
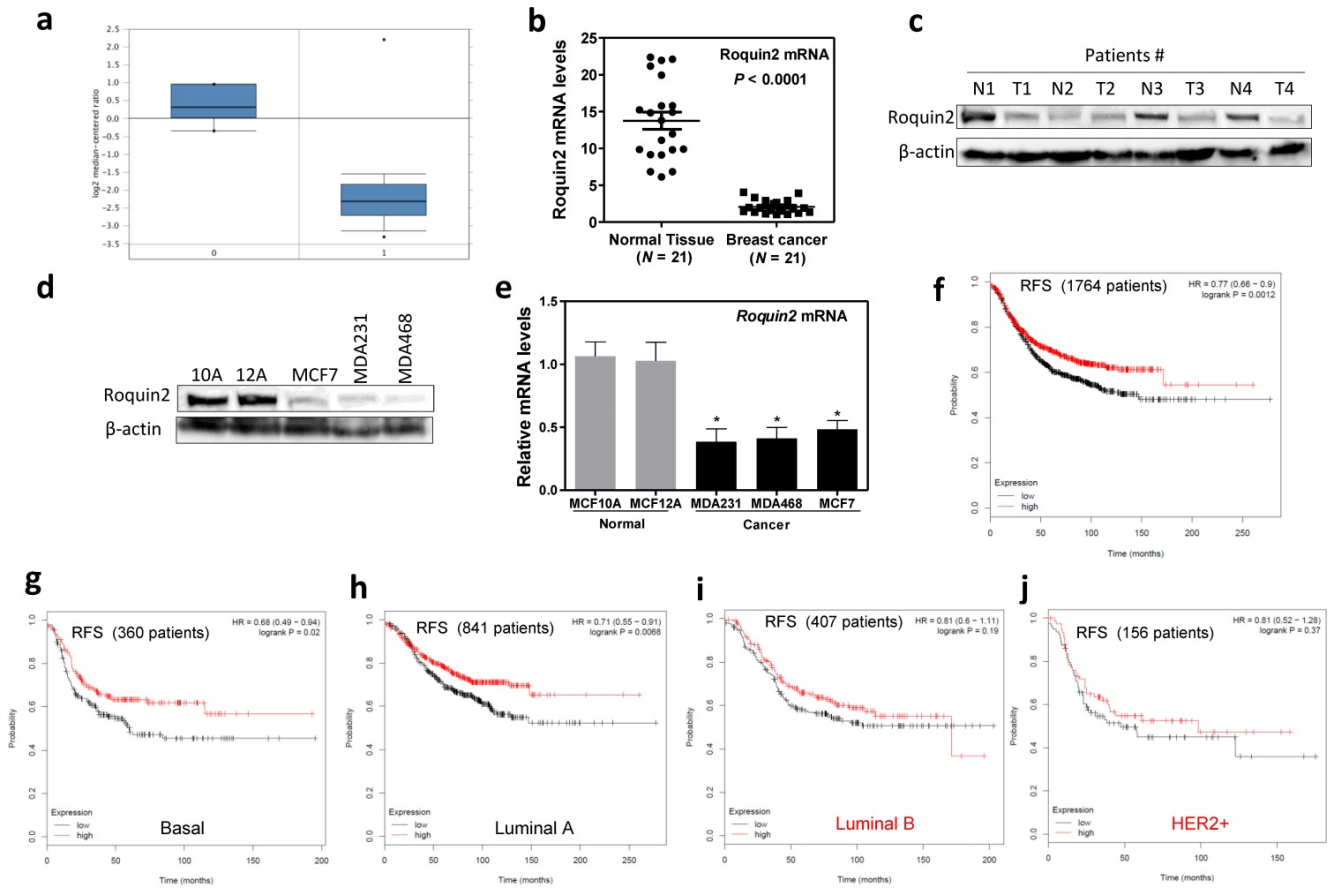
** Roquin2 is downregulated in breast cancer tissues and cells and associated with poor survival.** (A) Comparison of Roquin2 mRNA expression between normal (0) (*n* = 6) and invasive breast carcinoma (1) (*n* = 53). (B) *Roquin2* expression was measured by qPCR in human breast tumor specimens compared with surrounding “normal” breast tissue (*n* = 21 pairs) (*P* < 0.0001, unpaired Student's *t* test). (C) Roquin2 protein level was measured in human breast tumor tissues and normal mammary gland tissues. β-actin was used as a loading control. (D, E) Roquin2 protein and mRNA levels were measured respectively by Western blot and qPCR in human normal mammary gland epithelial cell lines and breast cancer cell lines. (F) Kaplan-Meier Relapse Free Survival curve of breast cancer patients with low and high tumor Roquin2 transcripts. (G-J) Kaplan-Meier Relapse Free Survival curves of basal (G), luminal A (H), luminal B (I), and Her2+ (J) breast cancer patients with low and high tumor Roquin2 transcripts. Statistical significance was determined by a two-tailed, unpaired Student's t test (*P < 0.05).

We investigate Roquin2 expression in breast cancer tissues by first comparing Roquin2 expression in various breast cancer tissues from TCGA breast cancer database (www.oncomine.org). Roquin2 mRNA levels were lower in invasive breast carcinoma (Fig. 1A), invasive ductal breast carcinoma, invasive lobular breast carcinoma (Supplemental Figure 1A), and different subtypes of breast cancers (Supplemental Figure 1B) (http://ualcan.path.uab.edu/index.html) compared to normal mammary tissue. We also found that Roquin2 mRNA expression was downregulated in 21 pairs of surgically removed breast tumors compared with their adjacent health tissue (Fig. 1B). Roquin2 protein expression was lower in randomly selected breast tumors compared to their adjacent healthy tissues (Fig. 1C). Additionally, Roquin2 protein (Fig. 1D) and mRNA (Fig. 1E) levels were downregulated in several commonly used breast cancer cell lines compared with normal mammary gland epithelial cells. Importantly, low Roquin2 expression in human breast tumor tissues correlated with poor patient Relapse Free Survival (Fig. 1F), Overall Survival and Distant Metastasis Free Survival (Supplemental Figure 1C, D) (http://dna00.bio.kyutech.ac.jp/PrognoScan/index.html) 28. We also found that the levels of Roquin2 in breast tumors had a significant correlation with patient survival in basal-like (Fig. 1G) and Luminal A (Fig. 1H) subtypes. Although no statistical significance was found between Roquin2 expression and patient survival in Luminal B and HER^2+^ breast cancer subtypes, they shared a similar trend (Fig. 1I, J). Taken together, these results demonstrated that Roquin2 expression is reduced in breast tumor tissues and Roquin2 levels correlates significantly with prognosis of breast cancer patients.

### Roquin2 inhibits human breast tumor angiogenesis in vitro and in vivo

**Figure 2:**
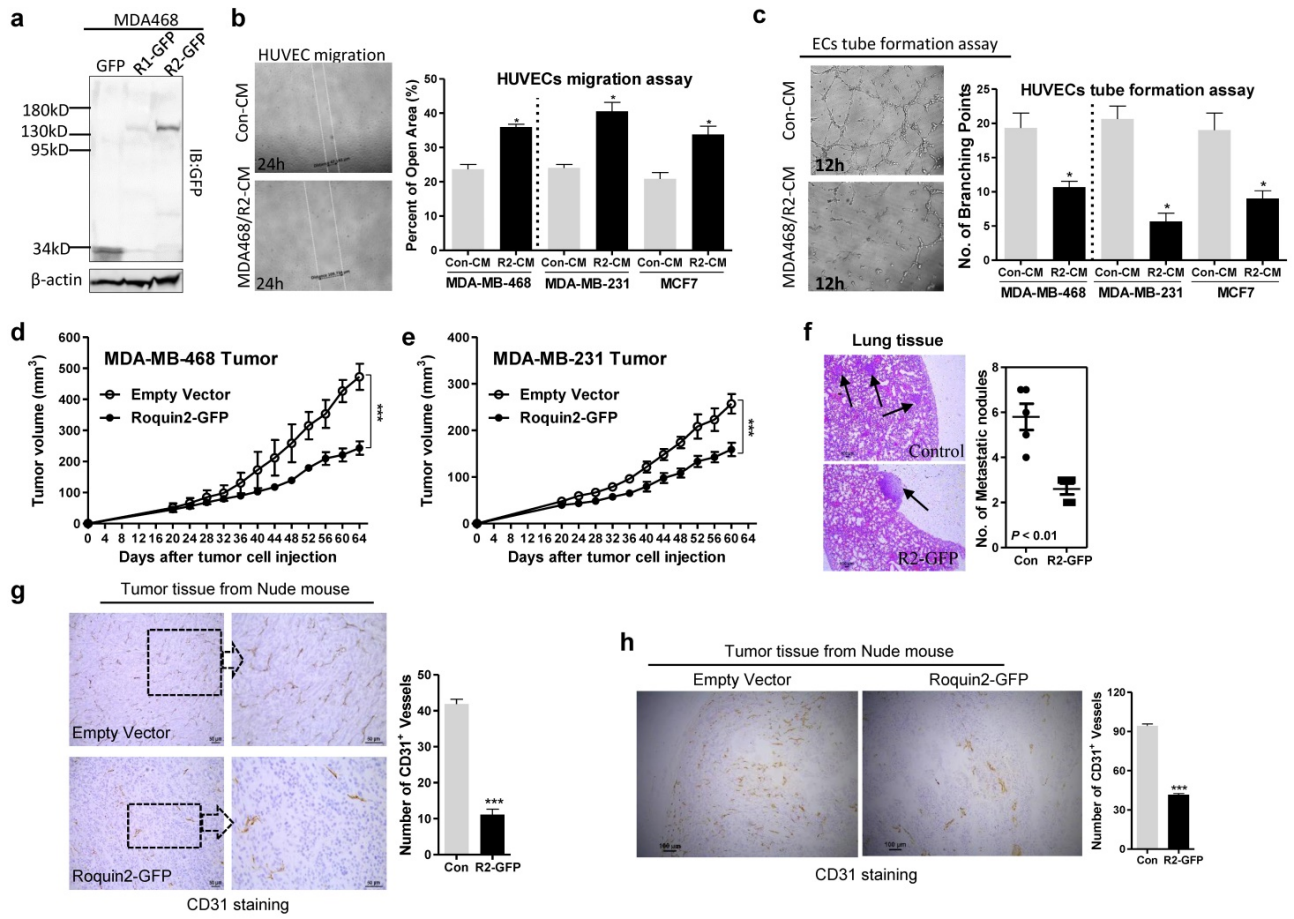
** Roquin2 inhibits breast tumor-induced angiogenesis *in vitro* and *in vivo*.** (A) Exogenous Roquin1/GFP and Roquin2/GFP fusion protein expression was detected by immunoblotting with anti-GFP antibody. (B) Left: Representative photographs of migrated HUVECs after treatment 24 hours with CMs from Roquin2-overexpressing MDA468/Roquin2 and its control MDA468/GFP cells. Right: Quantification of the percentage of open area of HUVECs treated with indicated tumor CMs in the wound-healing assay. (C) Left: Representative photographs of tube-formed HUVECs after treatment 12 hours with CMs from Roquin2-overexpressing MDA-MB-468 and its control MDA-MB-468/GFP cells. Right: Quantification of the number of branching points of HUVECs treated with indicated tumor CMs in the tube formation assay. (D, E) Tumor growth curves in nude mice received MDA-MB-468/Roquin2-GFP, MDA-MB-231/Roquin2-GFP and their control cells (*n* = 6/group), respectively. (F) H&E staining of lung tissue sections from nude mice bearing MDA-MB-468/GFP or MDA-MB-468/Roquin2-GFP tumors. Scale bar, 50µm. Quantification of metastatic nodules were shown in the right panel. (G) Left: Representative histological sections from MDA-MB-468/GFP and MDA-MB-468/Roquin2 tumors stained with a specific anti-CD31 antibody. Scale bar, 50µm. Right: Quantification of the number of CD31^+^ vessels per section. (H) Left: Representative histological sections from MDA-MB-231/GFP and MDA-MB-231/Roquin2 tumors stained with a specific anti-CD31 antibody. Scale bar, 50µm. Right: Quantification of the number of CD31^+^ vessels per section (right). Statistically significant results calculated with Student t-test are reported *P < 0.05; ***P < 0.001.

To investigate the role of Roquin2 in tumor angiogenesis, Roquin2 was first overexpressed in breast cancer cells and confirmed by immunoblots (Fig. 2A and Supplemental Figure 2A, B). The conditioned mediums (CMs) were then harvested to study the effect on human umbilical vein endothelial cell (HUVEC) migration and tube formation in vitro. CMs harvested from MDA-MB-468, MDA-MB-231, and MCF7 cells overexpressing Roquin2 could significantly suppress HUVEC migration (Fig. 2B) and tube formation (Fig. 2C), compared with their control groups. CMs from human lung cancer cells (A549) and human hepatocellular carcinoma cells (SMMC-7721) overexpressing Roquin2 could also inhibit HUVEC migration (Supplemental Figure 2C) and tube formation (Supplemental Figure 2D). The CMs from various types of tumor cells had no effect on the proliferation of endothelial cells themselves (Supplemental Figure 2E). To further evaluate the impact of Roquin2 expression on breast tumor angiogenesis *in vivo*, two breast tumor models were established. MDA-MB-468/Roquin2-GFP, MDA-MB-231/Roquin2-GFP cells and their control MDA-MB-468/GFP, MDA-MB-231/GFP cells subcutaneously injected into the back of nude mice. Tumor growth was significantly suppressed in mice injected with cells overexpressing Roquin2 in comparison to the control groups (Fig. 2D, E). Fewer metastatic white nodules and foci were found in the lungs of nude mice with tumors overexpressing Roquin2 compared to the control groups (Fig. 2F and Supplemental Figure 2F). Subsequently, tumor tissue slices from the two tumor models were stained by immunohistochemistry (IHC) with anti-CD31 (a typical marker of microvessels) antibody to evaluate the density of microvessels inside tumors. Density of microvessels was decreased in Roquin2-overexpressing tumor tissues compared with their control groups in both the tumor models (Fig. 2G, H). These findings further demonstrate that Roquin2 can inhibit breast tumor angiogenesis* in vitro* and *in vivo*, which might be a mechanism to against breast cancer progression.

### Roquin2 selectively downregulates mRNA of proangiogenic factors by targeting 3'UTRs

**Figure 3:**
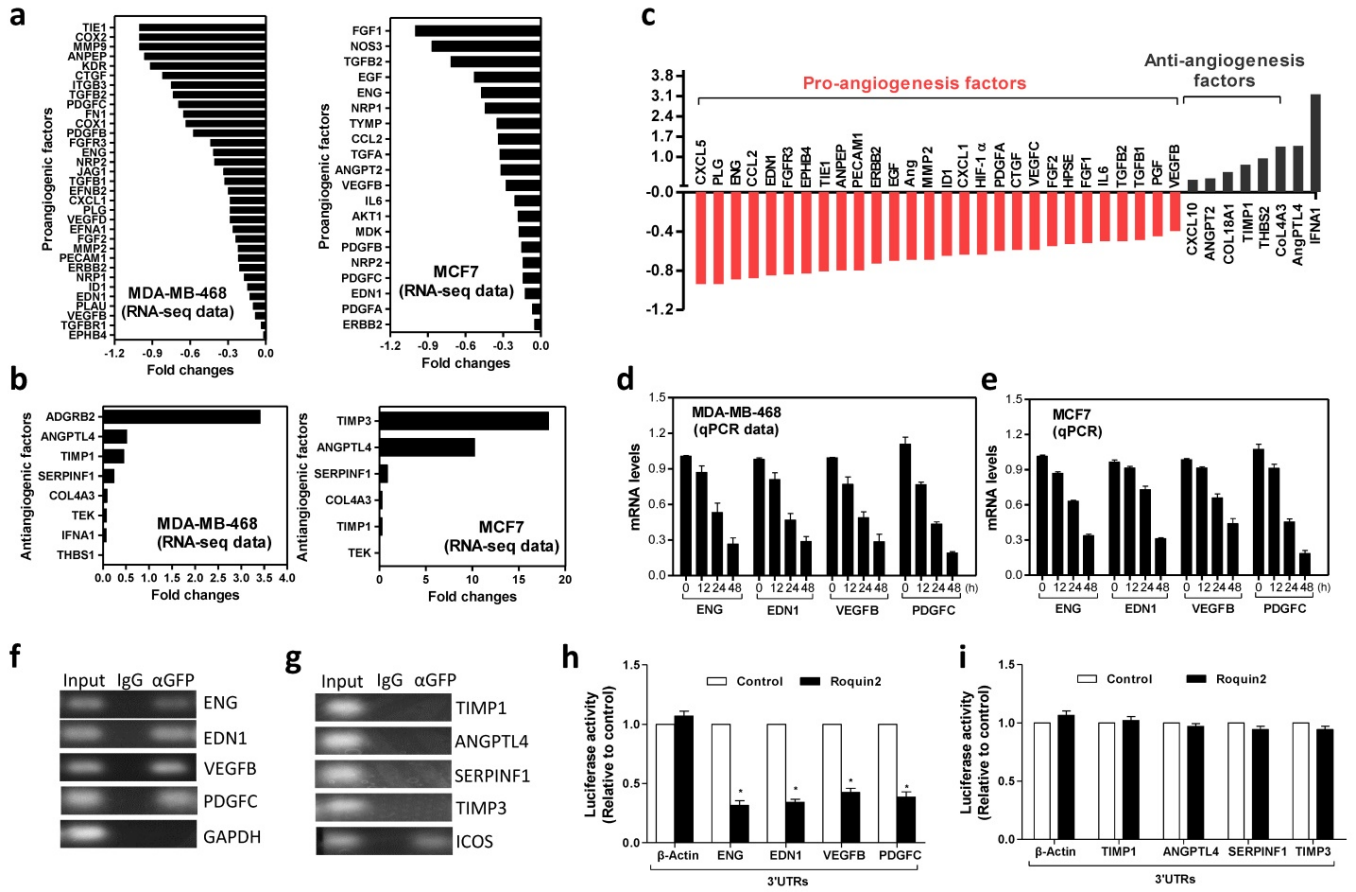
** Roquin2 selectively downregulates mRNA of proangiogenic factors by targeting 3'UTRs.** (A, B) RNA-seq data showing the proangiogenic factors mRNAs were downregulated (A), and the antiangiogenic factors mRNAs were upregulated (B) by Roquin2 in MDA-MB-468 and MCF7 cells, respectively. (C) Human angiogenesis pathway PCRArray showing Roquin2 downregulates proangiogenic factors genes and upregulates antiangiogenic factors genes in MDA-MB-468 cells. (D, E) qPCR confirming the proangiogenic factors mRNAs were downregulated by Roquin2 in a time dependent manner in MDA-MB-468 (D) and MCF7 (E) cells after infection with Roquin2-expressing adenovirus, respectively. (F, G) RNA-IP was performed using anti-GFP antibody or IgG in extraction of MDA-MB-468/Roquin2 cells. Proangiogenic (F) but antiangiogenic factors transcripts (G) were enriched by Roquin2. GAPDH transcript was used as a negative control and ICOS transcript served as a positive control. (H, I) Measurement of luciferase activities of reporters containing the 3'UTRs of indicated proangiogenic genes (H) and antiangiogenic genes (I), respectively. (Results shown represent the mean ± SEM of three independent experiments; *P < 0.05, unpaired Student's t-test).

Next, RNA-sequencing and PCRArray were performed to determine the effect of Roquin2 on the genes involved in tumor angiogenesis pathway. In Roquin2-overexpressing MDA-MB-468 and MCF7 cells, we found that proangiogenic factors mRNA, including *PDGFC*, *ENG*, *EDN1*, and *VEGFB*, were downregulated in two breast cancer cells, while the mRNA expression of antiangiogenic genes, including *TIMP1/3*, *SERPINF1*, and *ANGPTL4* were upregulated (Fig. 3A, B). Similar results were obtained in Roquin2-overexpressing A549 and SMMC-7721 cells (Supplemental Figure 3A-D). Moreover, the 'angiogenesis' term was significant enriched in the Gene Ontology (GO) analysis of regulated genes in breast cancer cells (Supplemental Figure 3E, F) (https://david.ncifcrf.gov/). These computational analyses further supported our experimental results. Detailed RNA-sequencing data are summarized in Supplemental Table 1. To further determine the link between Roquin2 and angiogenesis-related genes expression, we performed PCRArray after extracting total RNA from MDA-MB-468/Roquin2-GFP and its control cell MDA-MB-468/GFP. Clearly, we found 28 proangiogenic factors mRNAs were downregulated in the cells overexpressing Roquin2 (<-0.4-fold), and eight anti-angiogenic genes were upregulated (Fig. 3C and Supplemental Table 2). In order to validate the RNA-sequencing and PCRArray results, we measured the mRNAs levels of four downregulated proangiogenesis factors (*ENG*, *EDN1*, *VEGFB* and *PDGFC*) and four upregulated antiangiogenic factors (*TIMP1*, *SERPINF1*, *ANGPTL4* and *TIMP3*) at different time points after Roquin2 overexpression in MDA-MB-468 and MCF7 cells by qPCR. All four proangiogenic factors mRNA were downregulated in a time-dependent manner in breast cancer cells overexpressing Roquin2 (Fig. 3D, E). However, there was no time-dependence upregulation of anti-angiogenic mRNA levels (Supplemental Figure 3G, H). The results confirmed our RNA-sequencing and PCRArray data and suggesting that Roquin2 indeed modulates tumor angiogenesis by regulating the expression of angiogenesis-related genes.

To determine how Roquin2 controls these angiogenesis-related factors mRNA expression, RNA pull-down assay was performed with anti-GFP antibody and isotype IgG in MDA-MB-468 cells over-expressing Roquin2/GFP fusion protein. The mRNAs of proangiogenic factors, including *ENG*, *EDN1*, *VEGFB* and *PDGFC*, were detected by PCR. GAPDH was used the negative control (Fig. 3F). However, four anti-angiogenic factors mRNAs, including *TIMP1*, *ANGPTL4*, *SERPINF1* and *TIMP3*, could not be detected. *ICOS* was used as the positive control (Fig. 3G). These results indicated that Roquin2 could interact directly with the transcripts of proangiogenic factors in tumor cells. Previous studies have demonstrated that Roquin2 targets the 3'UTRs of mRNA 15. Therefore, we examined whether Roquin2 targets the 3'UTRs of proangiogenic mRNA. A series of reporter vectors containing the 3'UTRs of proangiogenic factors, including *ENG*, *EDN1*, *VEGFB* and *PDGFC*, were constructed (Supplemental Figure 3I). Luciferase assay was conducted by co-transfecting HEK293 cells with these reporters or empty vector with Roquin2 expression vector and then measured luciferase activity. Roquin2 could significantly reduce the luciferase activities of reporters containing the 3'UTRs of proangiogenic factor genes (Fig. 3H). However, Roquin2 had no effect on the activities of reporters containing the 3'TURs of antiangiogenic genes (Fig. 3I). These results demonstrate that Roquin2 might downregulate angiogenic mRNAs expression through targeting the 3'UTRs. We speculated that the upregulation of antiangiogenic genes might be a secondary effect of the angiogenesis imbalance created by Roquin2. In summary, our results demonstrated that Roquin2 selectively downregulated the expression of angiogenic mRNAs by targeting their 3'UTRs.

### Roquin2 destabilizes mRNAs of angiogenic factors via the ROQ domain

**Figure 4:**
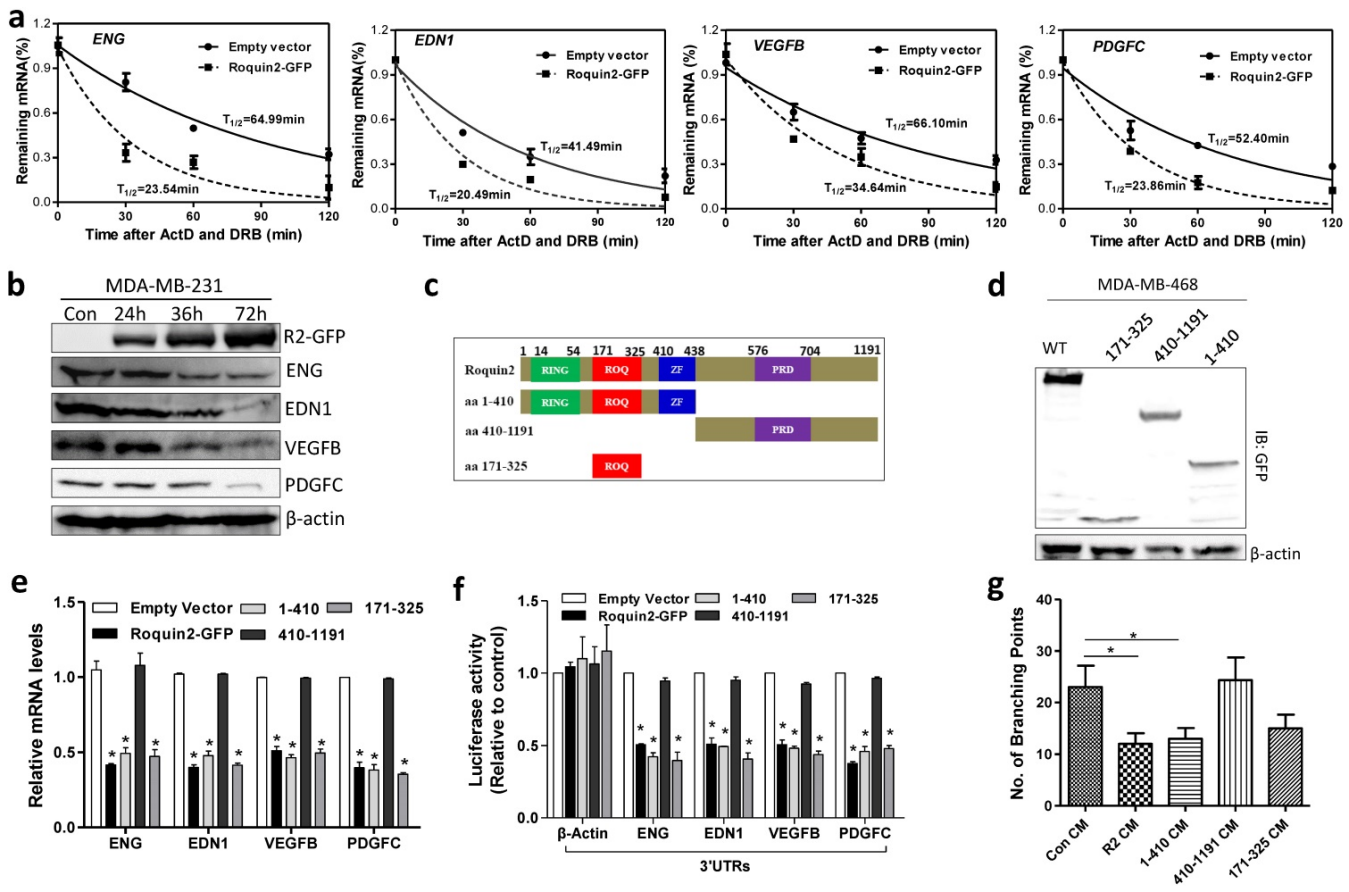
** Roquin2 destabilizes mRNAs of angiogenic factors via the ROQ domain.** (A) The half-lives of indicated angiogenic mRNAs were shortened by Roquin2 in MDA-MB-231 cells. (B) Cell lysates were isolated from the MDA-MB-231 cells infected with Roquin2-overexpressing adenovirus, followed by western blot analysis with GFP, ENG, EDN1, VEGFB, PDGFC, and β-actin antibodies. (C) Schematic representation of the domains in Roquin2, and their truncation mutants. (D) The expression of Roquin2 and its truncation mutants were confirmed by immunoblotting with anti-GFP antibody. (E) The expression of indicated angiogenic factors mRNAs were measured by qPCR after overexpressed Roquin2 and its mutants in MDA-MB-231 cells. (F) Relative luciferase activities of the indicated reporters were determined by the luciferase reporter assay. (G) Quantification of the number of branching points of HUVECs treated with indicated tumor CMs in the tube formation assay. Results shown represent the mean ± SEM of three independent experiments; unpaired Student's t-test, *P < 0.05.

Previous studies suggested that Roquin family proteins can destabilize mRNA 30. To verify this in our model, we overexpressed Roquin2 in MDA-MB-231 cells and then blocked *de novo* synthesis of mRNAs using ActD (5 μg/mL) and DRB (5 μg/mL), followed by the measurements of the remaining mRNAs at different time points. The half-lives of angiogenic mRNAs, including *ENG*, *EDN1*, *VEGFB* and *PDGFC*, were reduced approximately 2-fold in Roquin2-overexpressing tumor cells compared to the control group (Fig. 4A). However, Roquin2 overexpression had little effect on the half-lives of anti-angiogenic factor transcripts (Supplemental Figure 4A), suggesting that Roquin2 inhibits tumor angiogenesis through directly targeting and degrading proangiogenic mRNA but not anti-angiogenic gene transcripts. We found that the protein expression of ENG, EDN1, VEGFB and PDGFC was also reduced in a time-dependent manner after Roquin2 overexpression in MDA-MB-231 cells (Fig. 4B). The protein content of EDN1 and PDGFC in the CMs of MDA-MB-231 cells overexpressing Roquin2 were lower, as shown in the ELISA results (Supplemental Figure 4B).

To identify the functional domain of Roquin2 responsible for degrading angiogenic factor mRNAs, three truncated mutants were constructed: amino acid (aa) 1-410 (containing RING, ROQ, zinc finger domains), aa 410-1191 (containing PRD domain), and aa 171-325 (containing ROQ) (Fig. 4C) and confirmed by western blot (Fig. 4D). Wild-type (wt), and mutants aa 1-410 and aa 171-325 downregulated the mRNA expression of angiogenic genes (Fig. 4E) and decreased the luciferase activities of their 3'UTR reporters (Fig. 4F), while aa 410-1191 did not have any impact on the angiogenic genes levels. Moreover, CMs from MDA-MB-231 tumor cells transfected with wt, aa 1-410 and aa 171-325 mutants could inhibit HUVEC tube formation, however, CM from tumor cells transfected with aa 410-1191 mutant did not inhibit HUVEC tube formation (Fig. 4G and Supplemental Figure 4C). These results demonstrated that the ROQ domain of Roquin2 is critical for the destabilization of angiogenic mRNAs and the inhibition of tumor angiogenesis.

### Roquin2 targets the stem-loop structure in the 3'UTR of angiogenic genes for mRNA degradation

**Figure 5:**
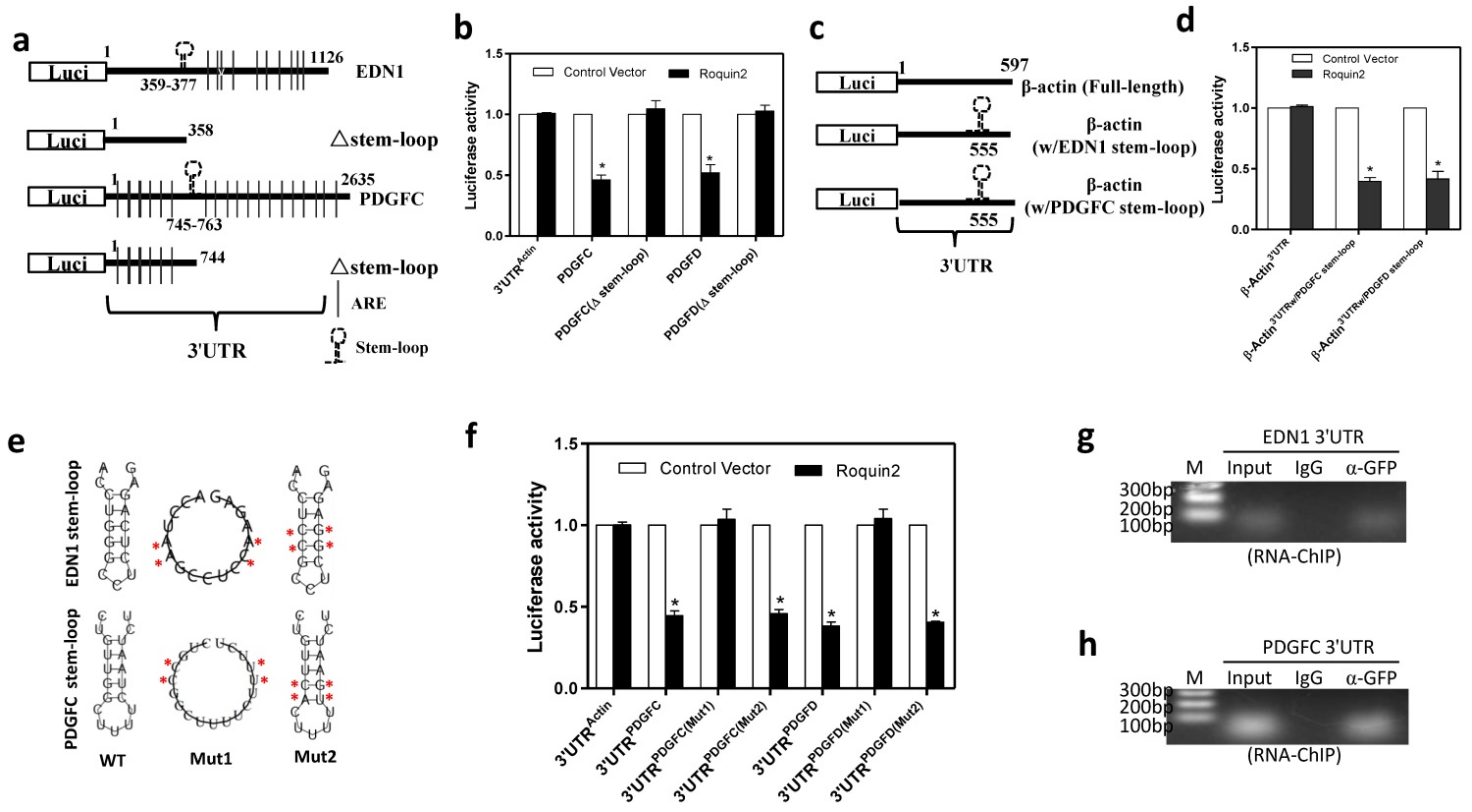
**Roquin2 targets the stem-loop structure in the 3'UTR of angiogenic genes for mRNA degradation.** (A) The diagram of the luciferase reporter constructs of EDN1 and PDGFC containing truncated 3'UTRs without the stem-loop structure. (B) Relative luciferase activities of the indicated reporters were determined by the luciferase reporter assay. (C) Schematic representation of the luciferase reporter constructs of human β-actin 3'UTR containing the stem-loop structure of *EDN1* (w/EDN1 stem-loop) or *PDGFC* (w/PDGFC stem-loop). (D) Relative luciferase activities of the indicated reporters were determined by the luciferase reporter assay. (E) The predicted stem-loop structures of EDN1 (top) and PDGFC (bottom) in their 3'UTRs and mutation strategy (asterisks indicate base substitution). Mutant1 becomes unable to form stem-loop structure (middle) and Mutant2 still forms a stem-loop structure (left). (F) Relative luciferase activities of the indicated reporters were determined by the luciferase reporter assay. (G, H) RNA-ChIP was conducted with genome fragments from MDA-MB-231/Roquin2-GFP cells. The pulled down 3'UTR sequences were amplified by PCR with primers flanking the putative stem-loop sequences. Results shown represent the mean ± SEM of at least three independent experiments. Unpaired Student's t-test, *P < 0.05.

It had been reported that Roquin1/2 targets the conserved stem-loop structure in 3'UTR for mRNA degradation 15. Here, we compared the 3'UTR sequence of all four angiogenic genes across different species and identified a conserved sequence, which could form a stem-loop structure when analyzing with the RNAfold webserver (Supplemental Figure 5A-D). To verify whether the stem-loop structures are necessary for Roquin2-mediated degradation of angiogenic mRNAs, we generated *EDN1* and *PDGFC* 3'UTRs stem-loop deletion constructs by deleting the 3'UTR sequence containing stem-loop structure (Fig. 5A). And then luciferase assay was performed by co-transfecting wild-type or deletion reporters with Roquin2 into HEK293 cells. The results showed that Roquin2 suppressed the luciferase activities of reporters with full-length *EDN1 and PDGFC* 3'UTRs but had no effect on luciferase activities of reporters without stem-loop structure (Fig. 5B). We also constructed luciferase reporters by inserting *EDN1 and PDGFC* stem-loop sequences into human *β-actin* 3'UTR (Fig. 5C) and performed luciferase assay. Roquin2 could reduce the luciferase activities of the reporters containing human *β-actin* 3'UTR sequence with *EDN1 and PDGFC* stem-loop structures (Fig. 5D). These results suggested that the stem-loop structures rather than AU-rich element (ARE) might be important for Roquin2-mediated angiogenic mRNAs degradation.

To define whether the RNA secondary conformation is required for Roquin2-mediated mRNA decay, we constructed two 3'UTR mutant reporters of EDN1 and PDGFC. The stem-loop structure was disrupted in mutant1 by replacing four nucleotides, whereas mutant2 preserved the stem**-**loop structure although substitution of four nucleotides in the stem and loop region (Fig. 5E). Luciferase assay results revealed that disruption of the stem-loop structure (mut1) made them completely resistant to Roquin2 (Fig. 5F), while mut2 was still sensitive to Roquin2 suppression, suggesting that the stem-loop structure rather than the linearized nucleotide sequence in the 3'UTRs is critical for Roquin2-mediated angiogenic mRNAs degradation. We further performed a modified RNA immunoprecipitation-chromatin immunoprecipitation (RIP-ChIP) assay to confirm the binding of Roquin2 with the stem-loop *in vivo*. *EDN1 and PDGFC* stem-loop sequences could be amplified in the groups pulled down with anti-GFP antibody but not the isotype IgG (Fig. 5G, H), suggesting that Roquin2 binds to the stem-loop in the 3'UTRs of angiogenic mRNAs inside breast cancer cells. Taken together, these results demonstrated that Roquin2 recognized the stem-loop structures in the 3'UTRs of angiogenic genes mRNAs for RNA destabilization.

### Roquin2 depletion increases angiogenic gene transcripts stability and promotes HUVEC migration and tube formation

**Figure 6:**
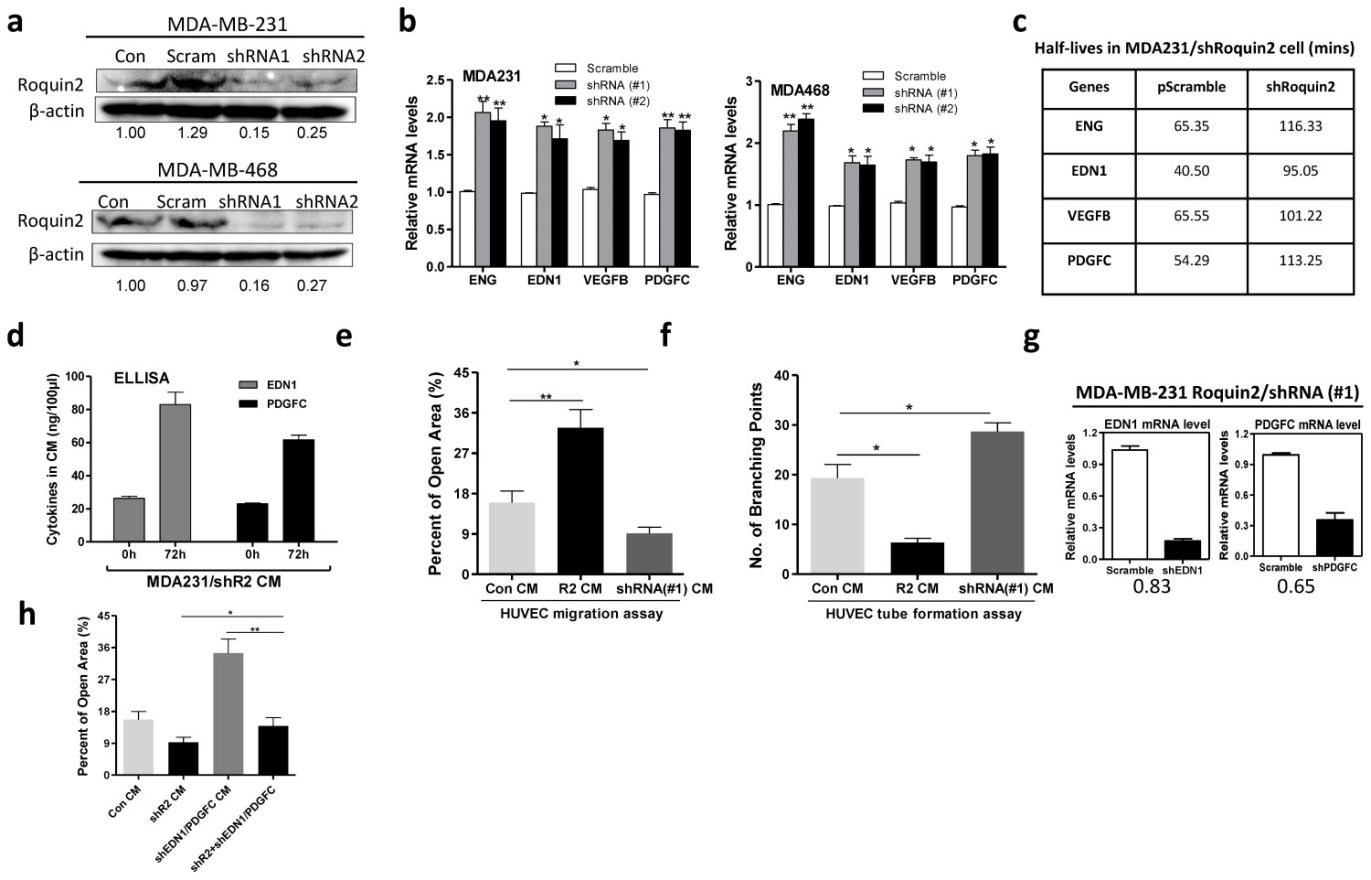
**Roquin2 depletion increases angiogenic gene transcripts stability and promotes tumor angiogenesis.** (A) Stable knockdown of Roquin2 protein expression by infecting Roquin2-shRNAs/lentivirus and scramble-shRNA/lentivirus in MDA-MB-231 and MDA-MB-468 cells, respectively, assessed by western blot. Western blot quantification was performed by ImageJ software. (B) The angiogenic factors mRNAs were measured in the MDA-MB-231 and MDA-MB-468 cells, respectively, after knockdown Roquin2 by qPCR. (C) The half-lives of indicated angiogenic genes were increased after Roquin2 knockdown in MDA-MB-231 cells. (D) ELISA quantification of EDN1 and PDGFC in serum-free culture medium of MDA-MB-231/shRoquin2 cells. Data are expressed in ng/100 μl of CM. (E) Quantification of open area of HUVECs treated with indicated tumor CMs in the wound-healing assay. (F) Quantification of the number of branching points of HUVECs treated with indicated tumor CMs in the tube formation assay. (G) MDA-MB-231/shRoquin2 cells were infected with scramble/lentivirus or shRNA-lentivirus targeting EDN1 and PDGFC, respectively. Total RNA extracted to measure mRNAs of EDN1 and PDGFC. (H) Quantification of open area of HUVECs treated with indicated tumor CMs. Unpaired Student's t-test, *P < 0.05, **P < 0.01.

To further confirm the effects of Roquin2 on tumor angiogenesis, we silenced Roquin2 expression with two shRNAs in MDA-MB-231 and MDA-MB-468 cells, respectively. Roquin2 protein expression was knocked down by about 85% and 75% by #1shRNA and #2shRNA, respectively (Fig. 6A). In line with the overexpression results, knocking down Roquin2 increased the mRNA levels of angiogenic genes, including *ENG, EDN1, VEGFB,* and *PDGFC* in breast cancer cells (Fig. 6B). However, the mRNA levels of antiangiogenic genes did not change compared with the scramble control (Supplemental Figure 6A). Roquin2 knockdown prolonged the half-lives of angiogenic mRNAs in MDA-MB-231 cells (Fig. 6C), but had little effect on the half-lives of antiangiogenic mRNAs (Supplemental Figure 6B). Furthermore, we also found that Roquin2 knockdown increased the protein levels of EDN1 and PDGFC in the CM harvested from MDA-MB-231 cells (Fig. 6D). Corresponding to the results of overexpression, the CMs from Roquin2 knockdown MDA-MB-231 cells significantly promoted HUVEC migration (Fig. 6E) and tube formation (Fig. 6F), further confirming the role of Roquin2 in tumor angiogenesis.

To verify whether the angiogenic genes were involved in Roquin2-mediated tumor angiogenesis, we silenced *EDN1 and PDGFC* using shRNA lentiviruses in MDA-MB-231/shRoquin2 cells (Fig. 6G). When *Roquin2* and *EDN1/PDGFC* were co-knockdown, HUVEC migration was restored to comparable to the scramble control group (Fig. 6H and Supplemental Figure 6C), suggesting that the angiogenic genes are indeed involved in Roquin2-mediated tumor angiogenesis. Besides, we also observed that overexpression of EDN1 and PDGFC could reverse the inhibitory effects of Roquin2 on tumor angiogenesis *in vitro* (Supplemental Figure 7A-D) and *in vivo* (Supplemental Figure 7E, F). Collectively, these results demonstrated that Roquin2 repression promotes breast tumor angiogenesis by increasing angiogenic mRNAs stability.

### Roquin2 suppresses breast tumor growth and metastasis progression

**Figure 7:**
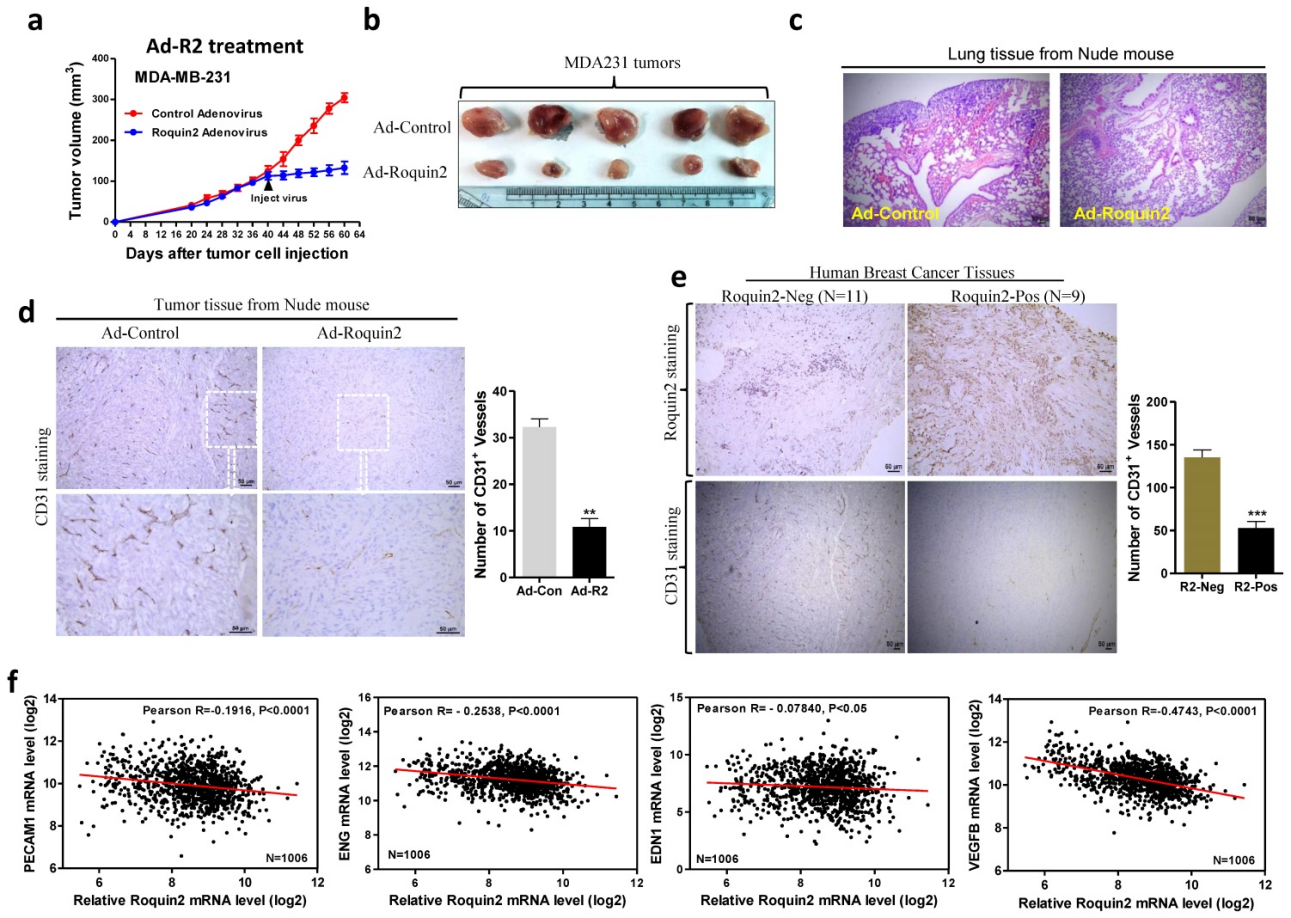
**Adenoviral expressing Roquin2 suppresses tumor progression and angiogenesis of the established breast tumors.** (A) Tumor growth curves after treatment with adenovirus. Black arrows indicated the time point of adenovirus injection. (B) Comparison of MDA-MB-231 tumors treated with control adenovirus (only express GFP protein) or Roquin2-expressing adenovirus. (C) H&E staining of lung sections of tumor-bearing mice treated with control adenovirus or Roquin2-expressing adenovirus, respectively. Scale bar, 50µm. (D) Left: Representative histological sections from tumors treated with control adenovirus or Roquin2-expressing adenovirus, tumors stained with a specific anti-CD31 antibody. Scale bar, 50µm. Right: Quantification of the number of CD31+ vessels per section. (Unpaired Student's t-test, **P < 0.01). (E) Left: Representative histological sections from human breast tumors stained with a specific anti-Roquin2 (up) and anti-CD31 (down) antibodies. Scale bar, 50µm. Right: Quantification of the number of CD31+ vessels per section. (Unpaired Student's t-test, ***P < 0.001). (F) Roquin2 expression was inversely correlated with the expression levels of angiogenic genes, including *PECAM1*, *ENG*, *EDN1*, and *VEGFB* in human breast cancer patients (*n* = 1006).

To simulate the clinical treatment of breast cancer, we prepared adenovirus expressing Roquin2/GFP fusion gene and the control virus (expressing GFP) to treat established MDA-MB-231 breast tumors in nude mice. When tumor mass reached a diameter of ~5 mm, 10^10^ pfu/100uL PBS adenovirus was injected every other day for five times in total (Supplemental Figure 8A). Two days after injection of Roquin2-expressing adenovirus, the growth of tumors was suppressed, while the tumors treated with control adenovirus continued growing (Fig. 7A). The sizes of tumors treated with Roquin2-expressing adenovirus were significantly smaller than those in the control group (Fig. 7B). Meanwhile, the lung tissue from nude mice bearing tumors treated with Roquin2-expressing adenovirus had significantly fewer metastatic foci compared with those of control group (Fig. 7C). Western blot was employed to confirm the expression of Roquin2/GFP fusion protein in tumor tissues (Supplemental Figure 8B). These results demonstrated that adenovirus-mediated *in vivo* expression of Roquin2 could suppress the growth and metastasis of breast tumor in nude mice. Moreover, the expression of *PECAM1*, *ENG*, *EDN1*, and *VEGFB* was decreased in MDA-MB-231 xenografts treated with Roquin2-expressing adenovirus (Supplementary Figure 8C). The density of CD31-positive microvessels in Roquin2-adenovirus treated tumors was reduced compared with those of the tumors treated with control adenovirus (Fig. 7D), which was in line with above *in vivo* results (Fig. 2) and further confirmed the inhibitory effects of Roquin2 on tumor angiogenesis. Furthermore, a total of 20 human breast tumor samples were analyzed by IHC staining with anti-Roquin2 antibody, of which nine samples were showed high Roquin2 expression (Roquin2-Positive) and eleven samples showed low Roquin2 expression (Roquin2-Negative). Notably, we found less CD31-positive microvessels were observed in Roquin2-Positive tumors than in Roquin2-Negative tumors (Fig. 7E). Moreover, there was a significant negative correlation between Roquin2 expression and the expression of angiogenic genes, including *PECAM1, ENG*, *EDN1*, *VEGFB* (Fig. 7F), and *ANG*, *TIE1*, *EPHB4* (Supplemental Figure 8D) in 1,006 human breast cancer samples, although no significant correlation between Roquin2 and PDGFC (data not shown) (Oncolnc.org/).

**Figure 8:**
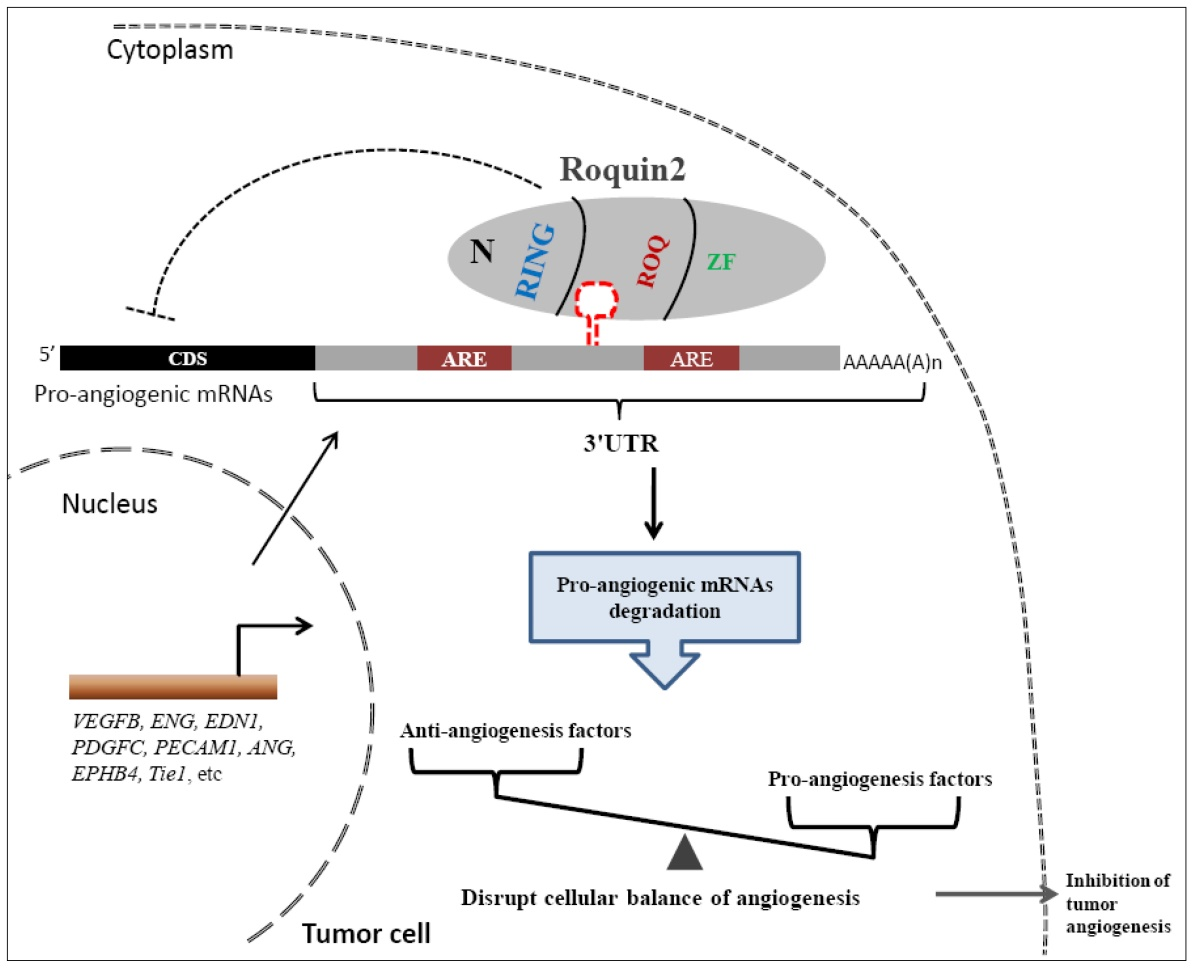
**A working model of Roquin2-mediated angiogenic mRNAs degradation and inhibition of tumor angiogenesis.** The mRNAs of proangiogenic factors were transported to the cytoplasm, where the Roquin2 protein recognizes these mRNAs by binding to the stem-loop structure rather than the AU-rich element (ARE) in the 3'UTRs via its ROQ domain and promotes the RNA degradation. As a consequence, the proangiogenic genes expression was inhibited by Roquin2, resulting in an imbalance of angiogenesis-related genes in tumor cells and the suppression of angiogenesis.

Finally, we proposed a model to illustrate the potential role of Roquin2 in the suppression of breast tumor angiogenesis (Fig. 8). Roquin2 preferentially targets angiogenic factors mRNA by binding to the stem-loop structures in the 3'UTRs and promotes degradation via its ROQ domain. As an RNA-binding protein located in the cytoplasm, Roquin2 could regulate tumor angiogenesis by tipping the balance of angiogenesis-related genes in tumor cells. In summary, these findings strongly demonstrated that Roquin2 is a novel breast cancer suppressor and Roquin2-angiogenic genes axis might be a promising target for breast cancer treatment.

## Discussion

Roquin1/2 has been shown to play an important role in suppressing autoinflammation by enhancing the mRNA decay of proinflammatory cytokines 31. In our previous study, we found that Roquin1 was able to suppress breast tumor progression by inducing cell cycle arrest of breast cancer cells 32. However, there are very few reports on the role of Roquin2 in tumor progression 26,27. In this study, we discovered the role of Roquin2 in regulating breast tumor angiogenesis confirmed by both *in vitro* and *in vivo* experiments. We showed that Roquin2 expression was suppressed in breast cancer tissues and cell lines and reduced Roquin2 correlates significantly with poor patient prognosis across the different subsets of breast cancer. These data demonstrated that Roquin2 is important for the progression of breast cancer.

Initially, we found that the target genes regulated by Roquin2 are also important for angiogenesis, including *TNF* 15, *IL6*, and *PTGS2* 31. Therefore, it is reasonable to think that Roquin2 may affect tumor angiogenesis in solid tumors. Indeed, Roquin2 can inhibit breast cancer cells-induced angiogenesis *in vitro* and *in vivo*. To our knowledge, there is no direct evidence showing Roquin2-mediated suppression of tumor angiogenesis in breast cancer. We also observed that Roquin2 was able to inhibit tumor angiogenesis induced by other types of cancer cells, including human lung cancer cells A549 and liver cancer cell SMMC-7721, suggesting that Roquin2 might has suppressive effect on other human cancers.

Tumor angiogenesis can be triggered once imbalance between angiogenic and antiangiogenic factors. Among Roquin2 targets, we chose four angiogenic genes (*ENG*, *EDN1*, *VEGFB*, and *PDGFC*) based on two criteria: 1) they are commonly downregulated in different types of cancer cells; and 2) they are among the most affected genes by Roquin2. Our RNA-sequencing and PCRArray data showed that these four genes typically fit the above criteria. The broad targets of Roquin2 in the angiogenesis pathway demonstrate that Roquin2 inhibits tumor angiogenesis in breast cancer cells through multiple molecular events.

We demonstrated that Roquin2 suppressed the expression of angiogenic genes at the post-transcriptional level by destabilizing their mRNAs. It has been known that the 3'UTR is crucial for the post-transcriptional regulation of genes. Many RNA-binding proteins such as MCPIP1 33, Roquin1 34 and human antigen R (HuR) 35 could regulate mRNA stability and gene expression by targeting 3'UTR. Our findings suggest that Roquin2 inhibits luciferase activity by the 3'UTRs of angiogenic genes. Studies have shown that the ROQ domain is crucial for Roquin2-mediated mRNA destabilization and immune regulation 17,18. Here, we showed that the ROQ domain was also necessary for the suppression of tumor angiogenesis by mutating the domains of Roquin2.

Roquin1/2 is known to recruit other deadenylases to the 3'UTR for mRNA degradation by targeting the stem-loop structure 15,21. Many elements responsible for mRNA turnover are located in the 3'UTR, such as AU-rich element (ARE), GU-rich element (GRE), and stem-loop structure 36,37. Our results demonstrate that the stem-loop structure is required for Roquin2-mediated degradation of angiogenic mRNAs. It is believed that Roquins bound a constitutive decay element (CDE) in the 3'UTR of TNFα mRNA and this CDE could fold into a stem-loop structure 15. Indeed, a conserved sequence was found among different specifies in all four angiogenic genes, respectively, and these sequences could fold into a stem-loop structure. No common stem-loop sequence was identified among the four angiogenic genes, further suggesting that Roquin2 recognizes the secondary stem-loop structure in 3'UTR but not linearized sequences, which is consistent with previous reports 20. Finally, Roquin2 expression correlated negatively with angiogenic mRNAs expression in human breast cancers, further confirming our basic research results. Collectively, our findings provided evidence for a new breast cancer suppressor which might be a promising molecular target for anti-angiogenic therapy in future.

## Supplementary Material

Supplementary figures and tables.

## Abbreviations

RBP: RNA-binding protein
3'UTR: 3' untranslated region
HUVEC: human umbilical vein endothelial cell
RING: Really Interesting New Gene
ZF: zinc finger
ROQ: ROQ domain containing RNA binding activity
ATCC: American Type Culture Collection
RPMI 1640: Roswell Park Memorial Institute 1640
shRNA: short hairpin RNA
DMEM: Dulbecco's Modified Eagle's Medium
RIP-ChIP: RNA immunoprecipitation-chromatin immunoprecipitation
VEGFB: vascular endothelial growth factor B
PDGFC: platelet derived growth factor C
ENG: endoglin
EDN1: endothelin-1
DRB: 5, 6-dichlorobenzimidazole riboside
ActD: Actinomycin D
ARE: AU-rich element
