# Supplementary material for: Roquin2 suppresses breast cancer progression by inhibiting tumor angiogenesis via selectively destabilizing proangiogenic factors mRNA
Source: Int J Biol Sci. 2021 Jul 13;17(11):2884–98. doi: 10.7150/ijbs.59891 (PMC8326130; doi:10.7150/ijbs.59891)
Supplement: Supplementary file 1 — Supplementary figures and tables. [file ijbsv17p2884s1.pdf]

Supplementary Information

Supplementary Figures

Supplementary Fig. S1. Roquin2 expression is reduced in breast cancers.

Supplementary Fig. S2. Roquin2 inhibits tumor cells-induced angiogenesis.

Supplementary Fig. S3. Roquin2 downregulates proangiogenic factors mRNA expression by targeting 3'UTR.

Supplementary Fig. S4. Roquin2 destabilizes mRNAs of proangiogenic factors via the ROQ domain.

Supplementary Fig. S5. Putative stem-loop structure in the 3'UTRs of proangiogenic genes.

Supplementary Fig. S6. Roquin2 depletion increases proangiogenic gene transcripts stability and promotes tumor angiogenesis.

Supplementary Fig. S7. EDN1 and PDGFC overexpression reversed the inhibitory effects of Roquin2 on tumor angiogenesis.

Supplementary Fig. S8. Adenoviral expressing Roquin2 suppresses tumor progression and angiogenesis of the established breast tumors.

Supplementary Tables (Tables S1 is provided as separate excel files):

Supplementary Table S1. RNA-seq analysis of human tumor cells overexpressing Roquin2.

Supplementary Table S2. PCRarray analysis of human tumor angiogenesis genes regulated by Roquin2.

Supplementary Table S3. List of PCR Primer Sequences used in this study.

Supplementary Figures

Supplementary Fig. S1

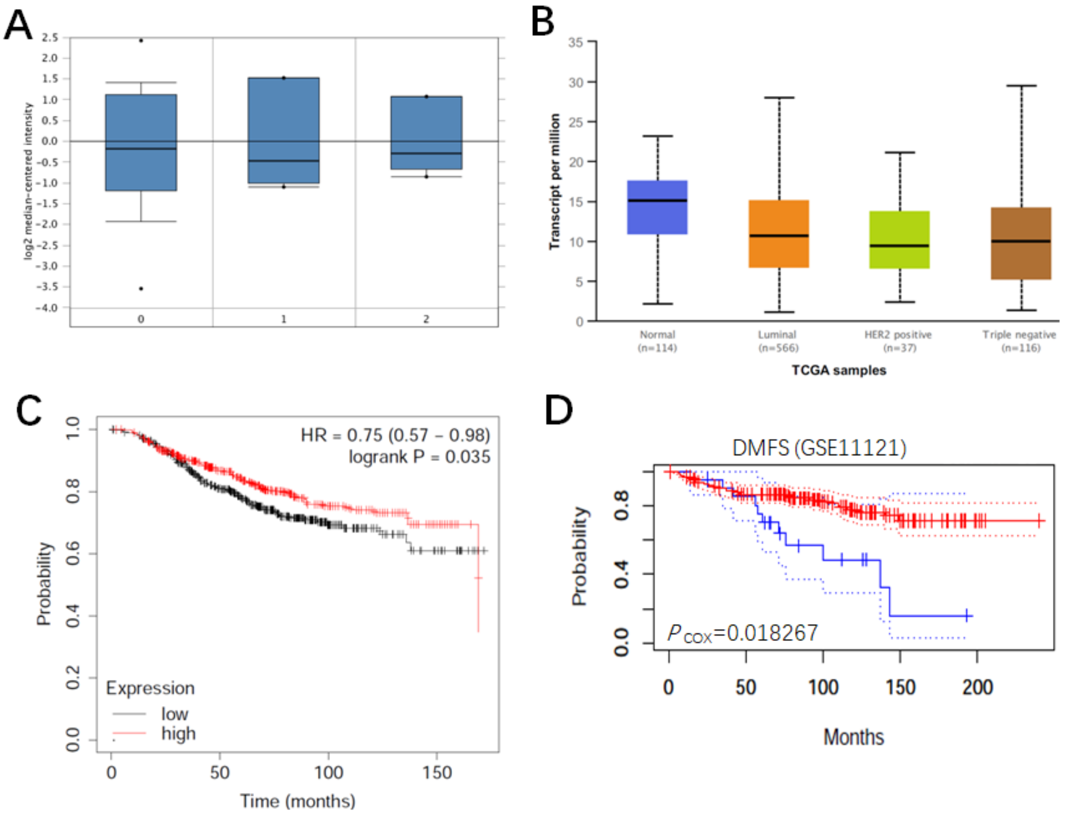

Supplementary Fig. S1. Roquin2 expression is reduced in breast cancers. (A) Comparison of Roquin2 mRNA expression between normal (0) (n = 20), invasive ductal breast carcinoma (1) (n = 5), and invasive lobular breast carcinoma (2) (n = 5). (http://www.oncomine.org/) (B) The expression levels of the Roquin2 gene were analyzed by the major subclasses of breast cancer based on the TCGA data. (http://ualcan.path.uab.edu/index.html) (C) Kaplan-Meier Overall Survival curve of breast cancer patients with low and high tumor Roquin2 transcripts. (http://kmplot.com/analysis/) (D) Kaplan-Meier Distant Metastasis Free Survival curve of breast cancer patients with low and high tumor Roquin2 transcripts. (http://dna00.bio.kyutech.ac.jp/PrognoScan/index.html)

Supplementary Fig. S2

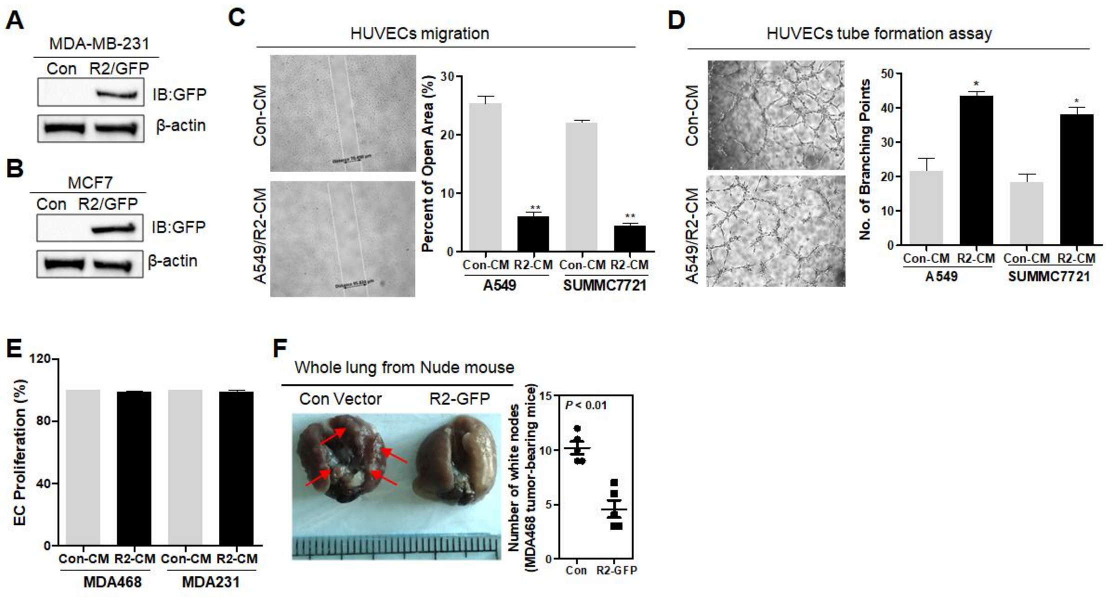

Supplementary Fig. S2. Roquin2 inhibits tumor cells-induced angiogenesis. (A, B) Cell lystates from MDA-MB-231 (A) and MCF7 (B) cells expressing Roquin2-GFP fusion protein or GFP were harvested for immunobloting analysis with anti-GFP and anti-β-actin antibodies. (C) Left: Representative photographs of migrated HUVECs after treatment 24 hours with CMs from Roquin2-overexpressing A549 and its control A549/GFP cells. Right: Quantification of the percentage of open area of HUVECs treated with indicated tumor CMs in the wound-healing assay. (D) Left: Representative photographs of tube-formed HUVECs after treatment 12 hours with CMs from Roquin2-overexpressing A549 and its control A549/GFP cells. Right: Quantification of the number of branching points of HUVECs treated with indicated tumor CMs in the tube formation assay. (E) CMs of Roquin2-expressing tumor cells had no effect on HUVECs proliferation expressed as percentage of untreated cells. (F) Left: Whole lung from nude mice bearing MDA-MB-468/GFP (control vector) or MDA-MB-468/Roquin2-GFP (R2-GFP) tumors was collected and compared. Right: Quantification of metastatic white nodules. Results shown represent the mean ± SEM of at least three independent experiments; *P < 0.05; **P < 0.01, unpaired Student's t-test.

Supplementary Fig. S3

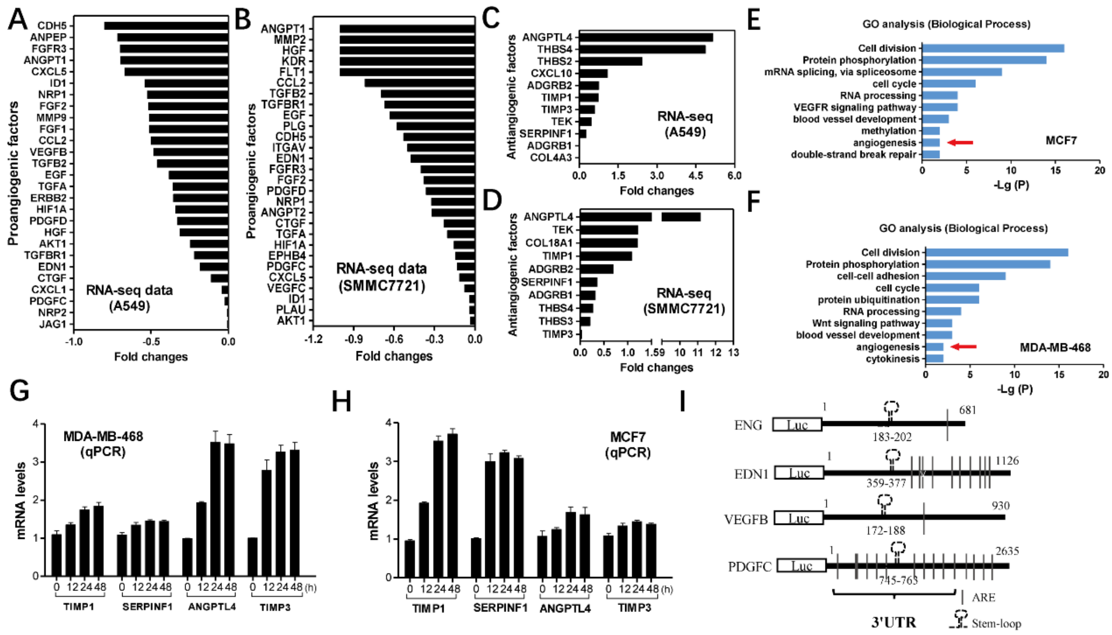

Supplementary Fig. S3. Roquin2 downregulates proangiogenic factors mRNA expression by targeting 3'UTR. (A-D) RNA-sequencing data showing the proangiogenic factors mRNA were downregulated by Roquin2 in A549 (A) and SMMCC-7721 (B) cells, and the antiangiogenic factors mRNAs were upregulated by Roquin2 in A549 (C) and SMMCC-7721 (D) cells. (E, F) ‘Angiogenesis’ term was significant enriched as a GO terms for all regulated genes by Roquin2 in MDA-MB-468 (E) and MCF-7 (F) cells. (G, H) qPCR showing the antiangiogenic factors mRNAs were upregulated by Roquin2 without a time dependent manner in MDA-MB-468 (G) and MCF7 (H) cells. (I) Schematic representation of the luciferase reporter constructs containing 3'UTRs sequences of *ENG*, *EDN1*, *VEGFB*, and *PDGFC*.

Supplementary Fig. S4

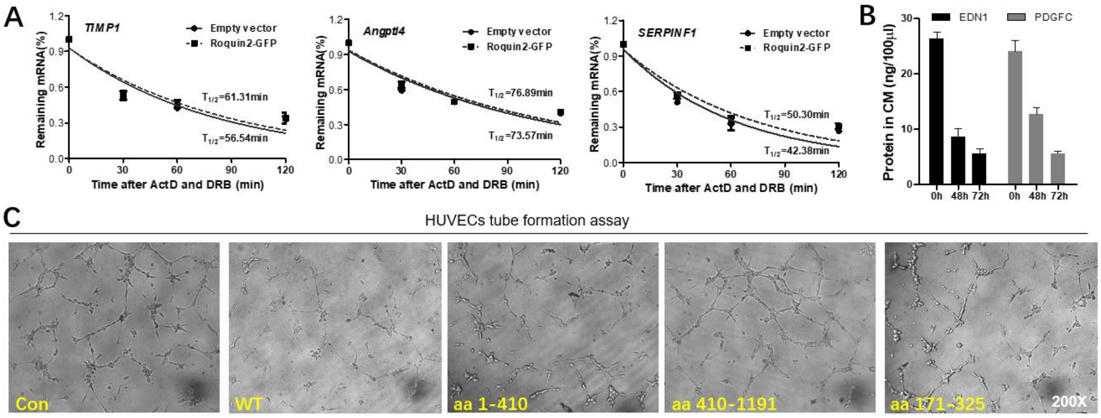

Supplementary Fig. S4. Roquin2 destabilizes mRNAs of proangiogenic factors via the ROQ domain. (A) Half-lives of indicated antiangiogenic factors mRNA were measured by qPCR in MDA-MB-231/Roquin2 cells. (B) ELISA quantification of EDN1 and PDGFC in serum-free culture medium of MDA-MB-231/Roquin2 cells at indicated times. Data are expressed in ng/100 μl of CM. (C) Representative photographs of tube-formed HUVECs treated with indicated tumor CMs in the tube formation assay.

Supplementary Fig. S5

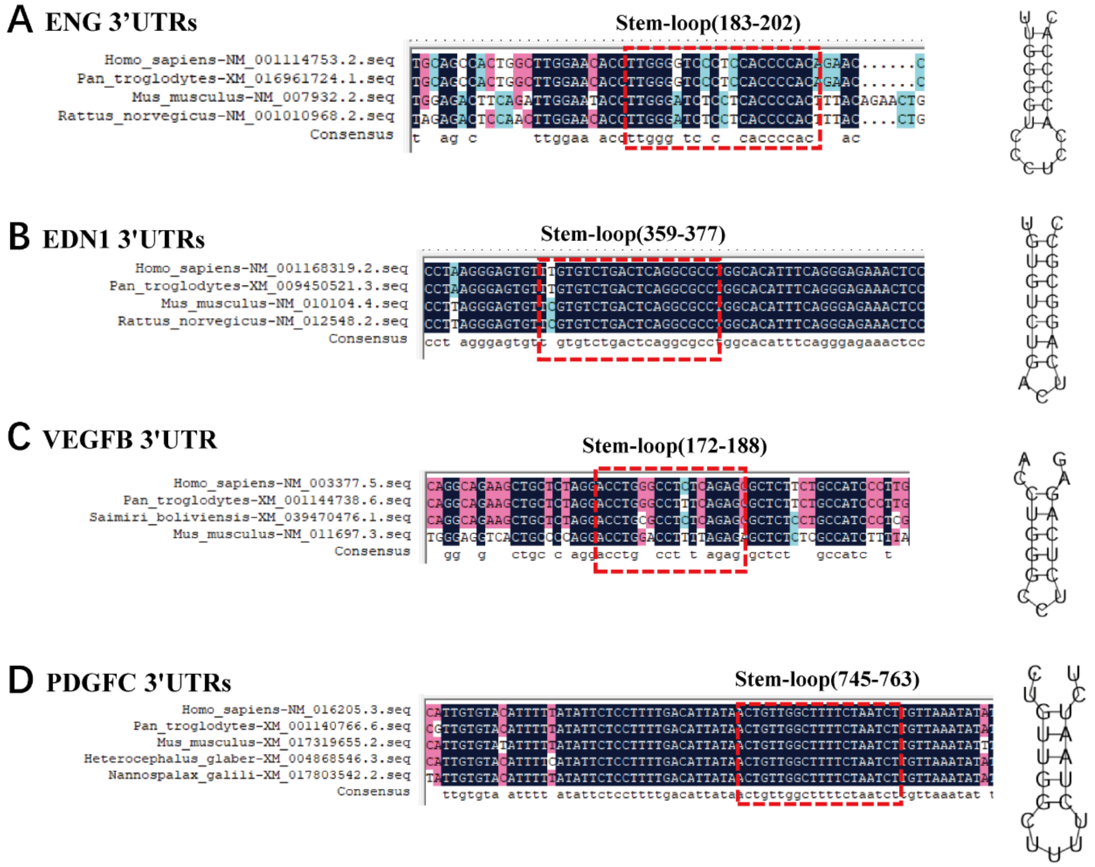

Supplementary Fig. S5. Putative stem-loop structure in the 3'UTRs of proangiogenic genes. (A-D) The 3'UTR sequences from different species for each proangiogenic gene, including *ENG* (A), *EDN1* (B), *VEGFB* (C), and *PDGFC* (D), was aligned using DNAMAN software. The stem-loop sequences were predicted by RNAfold web server to fold a secondary stem-loop structure (right) and indicated by red box.

Supplementary Fig. S6

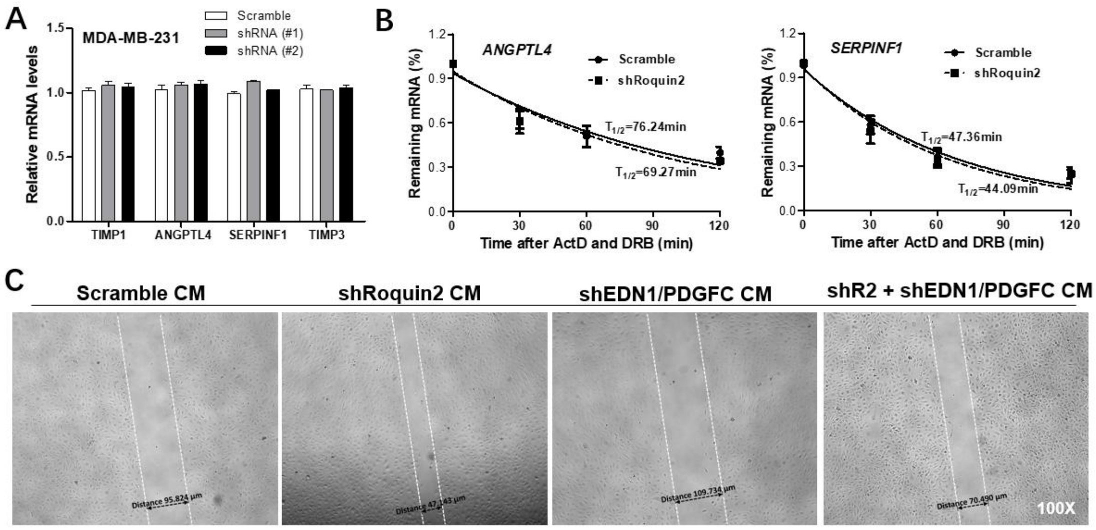

Supplementary Fig. S6. Roquin2 depletion increases proangiogenic gene transcripts stability and promotes tumor angiogenesis. (A) The antiangiogenic factors mRNAs were measured in the MDA-MB-231 cells after knocking down Roquin2 by qPCR. (B) The half-lives of *ANGPTL4* and *SERPINF1* were measured by qPCR in MDA-MB-231 cells after Roquin2 knockdown. (C) Representative photographs of migrated HUVECs treated with indicated tumor CMs in the wound-healing assay.

Supplementary Fig. S7

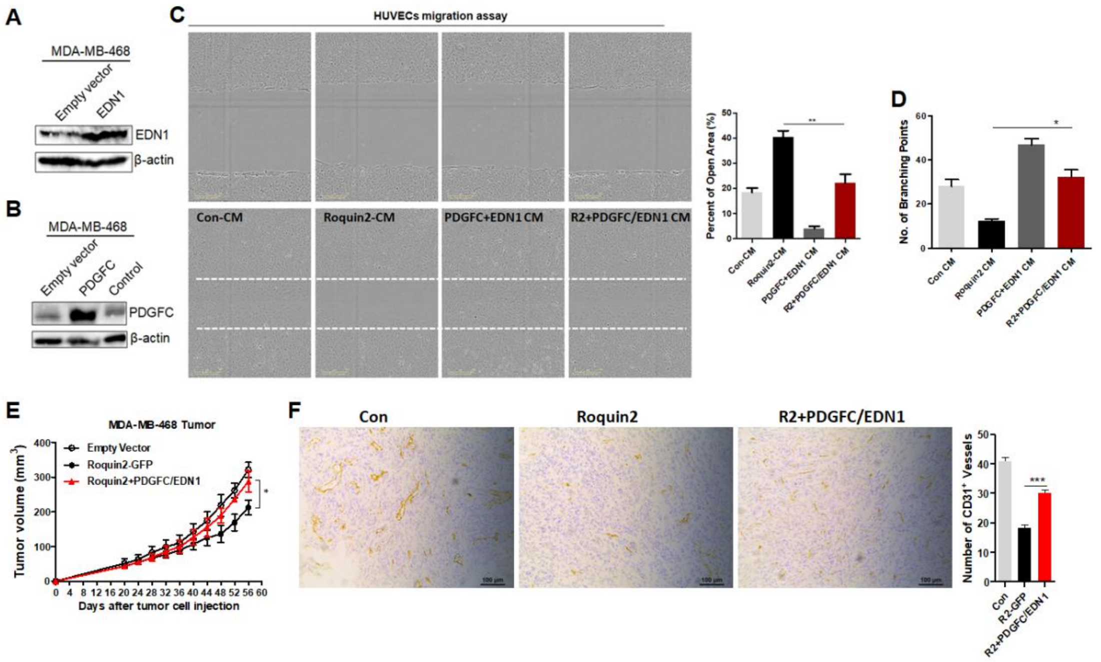

Supplementary Fig. S7. EDN1 and PDGFC overexpression reversed the inhibitory effects of Roquin2 on tumor angiogenesis. (A, B) EDN1 (A) and PDGFC (B) protein levels were measured in MDA-MB-468 cells by immunobloting with anti-EDN1, anti-PDGFC and anti-β-actin antibodies. β-actin was used as a loading control. (C) Left: Representative photographs of migrated HUVECs after treatment 24 hours with indicated CMs from MDA-MB-468 cells. Right: Quantification of the percentage of open area of HUVECs treated with indicated tumor CMs in the wound-healing assay. (D) Quantification of the number of branching points of HUVECs treated with indicated tumor CMs in the tube formation assay. (E) Tumor growth curves in nude mice received MDA-MB-468/Roquin2-GFP, MDA-MB-468/Roquin2-GFP/EDN1+PDGFC and their control cells (n = 6/group). (F) Left: Representative histological sections from MDA-MB-468/GFP, MDA-MB-468/Roquin2 and MDA-MB-468/Roquin2/EDN1+PDGFC tumors stained with a specific anti-CD31 antibody. Scale bar, 100 µm. Right: Quantification of the number of CD31$^+$ vessels per section. *P < 0.05; **P < 0.01; ***P < 0.001.

Supplementary Fig. S8

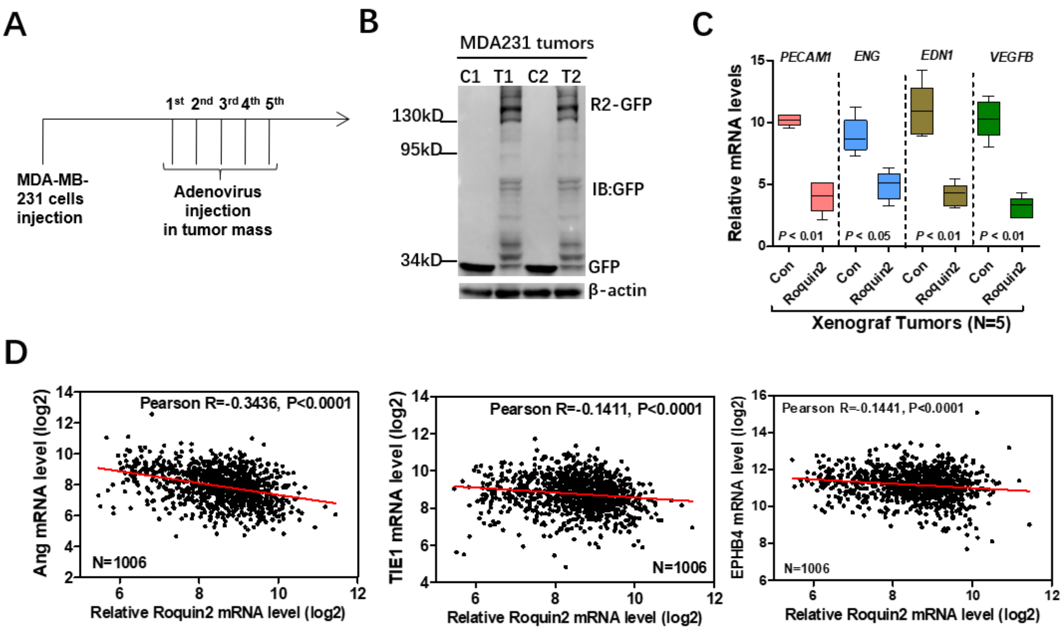

Supplementary Fig. S8. Adenoviral expressing Roquin2 suppresses tumor progression and angiogenesis of the established breast tumors. (A) Experimental flow chart for tumor treatment with Roquin2-expressing adenovirus *in vivo*. MDA-MB-231 cells (3×10^6cells/100 μL PBS) were injected into nude mice to establish tumor mass. Tumors were treated with adenovirus every one day when tumors grow to ~5mm in diameter. (B) Roquin2-GFP fusion protein expression in xenograft tumors was confirmed by immunoblotting with an anti-GFP antibody. (C) Measurement of the expression levels of *PECAM1*, *ENG*, *EDN1*, and *VEGFB* in Xenograft tumors. (D) Pearson's Correlation analysis between *Roquin2* and *angiogenin* (*ANG*), *Tie1*, and *EPHB4* expression levels in log2 values in human breast cancer patients (n = 1006).

Supplementary Table S2. PCRarray analysis of human tumor angiogenesis genes regulated by Roquin2

Gene Symbol	Gene Name	Control	Roquin2-Overexpression	fold change
AKT1	V-akt murine thymoma viral oncogene homolog 1	29.13	29.46	-0.09
ANG	Angiogenin, ribonuclease, RNase A family, 5	26.01	27.92	-0.69
ANGPT1	Angiopoietin 1	32.45	32.68	-0.02
ANGPT2	Angiopoietin 2	32.46	32.33	0.26
ANGPTL4	Angiopoietin-like 4	30.12	29.07	1.38
ANPEP	Alanyl (membrane) aminopeptidase	30.55	33.07	-0.80
BAI1	Brain-specific angiogenesis inhibitor 1	35.16	36.55	-0.56
CCL11	Chemokine (C-C motif) ligand 11	–	–	NA
CCL2	Chemokine (C-C motif) ligand 2	30.11	33.42	-0.88
CDH5	Cadherin 5, type 2 (vascular endothelium)	37.53	37.01	0.65
COL18A1	Collagen, type XVIII, alpha 1	31.45	31.09	0.47
COL4A3	Collagen, type IV, alpha 3 (Goodpasture antigen)	32.12	31.09	1.35
CTGF	Connective tissue growth factor	30.45	31.95	-0.59
CXCL1	Chemokine (C-X-C motif) ligand 1 (melanoma growth stimulating activity, alpha)	24.06	25.80	-0.64
CXCL10	Chemokine (C-X-C motif) ligand 10	32.14	32.06	0.21
CXCL5	Chemokine (C-X-C motif) ligand 5	22.21	26.44	-0.94
CXCL6	Chemokine (C-X-C motif) ligand 6 (granulocyte chemotactic protein 2)	–	-	NA
CXCL9	Chemokine (C-X-C motif) ligand 9	38.35	38.32	0.17
EDN1	Endothelin 1	21.61	24.54	-0.85
EFNA1	Ephrin-A1	28.38	28.34	0.18
EFNB2	Ephrin-B2	32.36	30.96	2.03
EGF	Epidermal growth factor	34.08	36.04	-0.70
ENG	Endoglin	32.07	35.46	-0.89
EPHB4	EPH receptor B4	27.15	29.88	-0.83
ERBB2	V-erb-b2 erythroblastic leukemia viral oncogene homolog 2, neuro/glioblastoma derived oncogene homolog (avian)	31.02	33.12	-0.73
F3	Coagulation factor III (thromboplastin, tissue factor)	30.83	29.82	1.31
FGF1	Fibroblast growth factor 1 (acidic)	35.12	36.37	-0.52
FGF2	Fibroblast growth factor 2 (basic)	25.15	26.51	-0.55
FGFR3	Fibroblast growth factor receptor 3	31.07	33.91	-0.84
FIGF	C-fos induced growth factor (vascular endothelial growth factor D)	33.35	33.25	0.23
FLT1	Fms-related tyrosine kinase 1 (vascular endothelial growth factor/vascular permeability factor receptor)	–	-	NA
FN1	Fibronectin 1	27.88	26.94	1.20
HGF	Hepatocyte growth factor (hepapoietin A; scatter factor)	34.10	34.43	-0.08
HIF1A	Hypoxia inducible factor 1, alpha subunit (basic	26.12	27.79	-0.64

	helix-loop-helix transcription factor)			
HPSE	Heparanase	29.01	30.31	-0.53
ID1	Inhibitor of DNA binding 1, dominant negative helix-loop-helix protein	22.12	23.84	-0.65
IFNA1	Interferon, alpha 1	34.87	33.01	3.16
IFNG	Interferon, gamma	–	-	NA
IGF1	Insulin-like growth factor 1 (somatomedin C)	33.11	33.13	0.13
IL1B	Interleukin 1, beta	32.25	33.10	-0.36
IL6	Interleukin 6 (interferon, beta 2)	28.85	30.03	-0.50
IL8	Interleukin 8	27.02	27.37	-0.10
ITGAV	Integrin, alpha V (vitronectin receptor, alpha polypeptide, antigen CD51)	29.48	30.30	-0.35
ITGB3	Integrin, beta 3 (platelet glycoprotein IIIa, antigen CD61)	36.25	34.55	2.73
JAG1	Jagged 1	28.03	28.30	-0.05
KDR	Kinase insert domain receptor (a type III receptor tyrosine kinase)	–	-	NA
LECT1	Leukocyte cell derived chemotaxin 1	–	-	NA
LEP	Leptin	33.76	34.02	-0.04
MDK	Midkine (neurite growth-promoting factor 2)	33.55	34.32	-0.33
MMP14	Matrix metallopeptidase 14 (membrane-inserted)	–	-	NA
MMP2	Matrix metallopeptidase 2 (gelatinase A, 72kDa gelatinase, 72kDa type IV collagenase)	30.11	31.99	-0.69
MMP9	Matrix metallopeptidase 9 (gelatinase B, 92kDa gelatinase, 92kDa type IV collagenase)	30.05	29.12	1.19
NOS3	Nitric oxide synthase 3 (endothelial cell)	–	-	NA
NOTCH4	Notch 4	35.33	33.55	2.94
NRP1	Neuropilin 1	26.15	26.06	0.22
NRP2	Neuropilin 2	28.25	28.09	0.28
PDGFA	Platelet-derived growth factor alpha polypeptide	25.14	26.65	-0.60
PECAM1	Platelet/endothelial cell adhesion molecule	30.63	33.18	-0.80
PF4	Platelet factor 4	34.03	34.71	-0.28
PGF	Placental growth factor	28.35	29.42	-0.45
PLAU	Plasminogen activator, urokinase	28.60	29.46	-0.37
PLG	Plasminogen	34.26	38.48	-0.94
PROK2	Prokineticin 2	–	-	NA
PTGS1	Prostaglandin-endoperoxide synthase 1 (prostaglandin G/H synthase and cyclooxygenase)	36.45	35.03	2.07
S1PR1	Sphingosine-1-phosphate receptor 1	35.98	35.24	0.92
SERPINE1	Serpin peptidase inhibitor, clade E (nexin, plasminogen activator inhibitor type 1), member 1	27.57	27.77	0
SERPINF1	Serpin peptidase inhibitor, clade F (alpha-2 antiplasmin, pigment epithelium derived factor), member 1	32.06	32.53	-0.17
SPHK1	Sphingosine kinase 1	33.22	31.10	3.99

TEK	TEK tyrosine kinase, endothelial	37.33	37.31	0.16
TGFA	Transforming growth factor, alpha	31.47	31.76	-0.06
TGFB1	Transforming growth factor, beta 1	25.12	26.30	-0.49
TGFB2	Transforming growth factor, beta 2	28.02	29.22	-0.50
TGFBR1	Transforming growth factor, beta receptor 1	30.59	30.84	-0.03
THBS1	Thrombospondin 1	29.02	30.60	-0.62
THBS2	Thrombospondin 2	36. 83	36.06	0.95
TIE1	Tyrosine kinase with immunoglobulin-like and EGF-like domains 1	37.43	40	-0.81
TIMP1	TIMP metallopeptidase inhibitor 1	27.81	27.22	0.73
TIMP2	TIMP metallopeptidase inhibitor 2	30.34	30.56	0.01
TIMP3	TIMP metallopeptidase inhibitor 3	33.41	34.13	-0.30
TNF	Tumor necrosis factor	33.14	33.76	-0.25
TYMP	Thymidine phosphorylase	28.62	28.32	0.41
VEGFA	Vascular endothelial growth factor A	26.54	26.04	0.62
VEGFB	Vascular endothelial growth factor B	24.41	25.33	-0.40
VEGFC	Vascular endothelial growth factor C	27.32	28.81	-0.59
ACTB	Actin, beta	21.28	22.99	
B2M	Beta-2-microglobulin	24.44	23.87	
GAPDH	Glyceraldehyde-3-phosphate dehydrogenase	19.85	20.05	
HPRT1	Hypoxanthine phosphoribosyltransferase 1	28.76	28.70	
RPLP0	Ribosomal protein, large, P0	18.66	19.54	
HGDC	Human Genomic DNA Contamination			
RTC	Reverse Transcription Control			
RTC	Reverse Transcription Control			
RTC	Reverse Transcription Control			
PPC	Positive PCR Control	21.39	21.74	
PPC	Positive PCR Control	21.58	21.73	
PPC	Positive PCR Control	21.54	21.91	

Supplementary Table S3. List of PCR Primer Sequences

Gene name	Primer (sense)	Primer (anti-sense)
HuRoquin2 (qPCR)	AAGACCATGTGATTCTGGAGGAGCAA A	ACCATCTTCCCATTTGCAACAGGTTTT
huGAPDH (qPCR)	ACCTGCCAAATATGATGACATCAAGAA GGT	GTTGCTGTAGCCAAATTCGTTGTCATACC
HuPDGFC (qPCR)	AGGCGGAATCCAACCTGAGTAGTAAAT	CATCTTCTGGGTCTTCAAGCCCAAATC
HuVEGFB(qPCR)	ATG GAT AGA TGT GTA TAC TCG CGC TAC C	TTT TTA GGT CTG CAT TCA CAC TGG CTG
HuENG (qPCR)	TCAAGATCTTTCCAGAGAAAAACATTC GTGG	AACAAGCTCTTTCTTTAGTACCAGGGTCAT
HuEDN1 (qPCR)	ATGGATAAAGAGTGTGTCTACTTCTGC CA	CTGAGTTCTTTTCCTGCTTGGCAAAAATT
HuTIMP1 (qPCR)	ATACCAGCGTTATGAGATCAAGATGAC CA	AAAACTGCAGGTAGTGATGTGCAAGAG
HuSERPINF1 (qPCR)	TGGTACCTATAAGGAGCTCCTTGACAC G	ATCTCATCGGGAATTTCCTTTGTGGAC
HuANGPTL4 (qPCR)	TGCAGACACAACTCAAGGCTCAGAAC A	ATCGTGGCGCCTCTGAATTACTGT
HuTIMP3 (qPCR)	GATGAAGATGTACCGAGGCTTCACCAA	GGTGATACCGATAGTTCAGCCCCTT
HuICOS (qPCR)	TTTAACAGGAGAAATCAATGGTTCTGC CAA	GGATAACTGAGAATGGCAGAATTTCAGACTC
HuPDGFC (full-length 3UTR)	TTCTAGACCGCATCACCACCAGCAGCT CTTGCCCAGA	GGCCGGCCTGACATTTAAGGCTTGCTTTTATT TTAGTA AACAT
HuVEGFB (full-length 3UTR)	TTCTAGACAGCTCAACCCAGACACCTG CAGGTGCC	GGCCGGTGC CTG GCA GGA AGA ACA AGT GTT CTT TTC TAT TA
HuENG (full-length 3UTR)	TTCTAGACCGCGCTCGCCCAGCAGGA GAGACTGAGCA	GGCCGGCCCATGGTCTGATTTATTGGTGGTGA ATACACA
HuEDN1 (full-length 3UTR)	TTCTAGACAGACCTTCGGGGCCTGTCT GAAGCCATAG	GGCCGGCCTACAGTAAGGAAAAAAATATTTAT TTTCTA AAGT
HuTIMP1 (full-length 3UTR)	TTCTAGAATCCTGCCCGGAGTGGAAGC TGAAGCCT	GGCCGGCCTGCTGGGTGGTAACTCTTTAT TTCATTGTC
HuANGPTL4 (full-length 3UTR)	TTCTAGACGTCCTGGCTGGGCCTGGTC CCAGGCCCAC	GGCCGGCCTGTTCTGAGGTTGCTTTTATTCCA AGA ACTCT
HuSERPINF1 (full-length 3UTR)	TTCTAGATATCCCAGTTTAATATTCCA ATACCCTAGA	GGCCGGCCTAACAGAAGTTAGGGATA AAGCTCTTTTAT
HuTIMP3 (full-length 3UTR)	TTCTAGAGCGCCAGACCCTGCCCCACC TCACTTCCCT	GGCCGGCCGTGATAGAAATAAAACCACTTTAC TGTTTA
Huβ-actin (full-length 3UTR)	TTCTAGAGCGGACTATGACTTAGTTGC GTTACACCCTTTCTTGACAA	GGCCGGCCTTTAAGGTGTGCACTTTTATTCAA CTGGTCTCAAG
HuEDN1 (full-length)	TTCTAGAATGGATTATTTGCTCATGAT TTTCTCTCTGC	GGCCGGTCACCAATGTGCTCGGTTGTGGGTC ACATAA
HuPDGFC (full-length)	TTCTAGAATGAGCCTCTTCGGGCTTC TCCTGCTGACAT	GGCCGGCTATCCTCCTGTGCTCCCTCTGCAC ACA

Huβ-actin (w/PDGFC stem-loop)	CTGTTGGCTTTTCTAATCTACTGACTTG AGACCAGTTGAAT	GTACAGGTAAGCCCTGGCTGCCT
Huβ-actin (w/PDGFD stem-loop)	AAAGATGAGGCTTTCTTAACTGACTTG AGACCAGTTGAAT	GTACAGGTAAGCCCTGGCTGCCT
HuPDGFC (truncated 3UTR)	TTCTAGACCGCATCACCACCAGCAGCT CTTGCCCAGA	GGCCGGCCTTATAATGTCAAAAGGAGAATATA AAAAT
HuEDN1 (truncated 3UTR)	TTCTAGACAGACCTTCGGGGCCTGTCT GAAGCCATAG	GGCCGGAACACTCCCTTAGGACCTTCGTCAG AAACT
HuEDN1 (3UTR-mut1)	ATTATAAACCTAAGCCTCCAAGAGTGT TAAA	GTCAAAAGGAGAATATAAAAATGTACACAAT GA AAC
HuEDN1 (3UTR-mut2)	ATTATAAACCTCCGCCTCGGAGAGTGT TAAA	GTCAAAAGGAGAATATAAAAATGTACACAAT GA AAC
HuPDGFC (3UTR-mut1)	5’ATTATAACTGCCGGCTTTTCTTTTCTT GTTAAA -3'	GTCAAAAGGAGAATATAAAAATGTACACAAT GA AAC
HuPDGFC (3UTR-mut2)	5’ATTATAACTGTTCACTTTTTGAATCTT GTTAAA-3'	GTCAAAAGGAGAATATAAAAATGTACACAAT GA AAC
HuPDGFC (RNA-ChIP)	5’-GTACATTTTTATATTCTCCTTTTGAC-3	5’-GCTAAAAATAGTTGATCTAAGTTGTC-3'
HuEDN1 (RNA-ChIP)	5’AGATTCCACACAGGGGTGGAGTTTCT -3’	5’-GTGGACTTTGGAGTTTCTCCCTGAAA-3'
